# Low-Temperature Plasma Short Exposure to Decontaminate Peri-Implantitis-Related Multispecies Biofilms on Titanium Surfaces *In Vitro*

**DOI:** 10.1155/2022/1549774

**Published:** 2022-10-26

**Authors:** Beatriz H. D. Panariello, Drashty P. Mody, George J. Eckert, Lukasz Witek, Paulo G. Coelho, Simone Duarte

**Affiliations:** ^1^Department of Biomedical Sciences and Comprehensive Care, Indianapolis, IN, USA; ^2^Department of Cariology, Operative Dentistry and Dental Public Health Indianapolis, Indianapolis, IN, USA; ^3^Department of Biostatistics and Health Data Science, Indiana University School of Medicine, IN, USA; ^4^Department of Biomaterials, New York University College of Dentistry, New York, NY, USA; ^5^American Dental Association Science and Research Institute, Chicago, IL, USA

## Abstract

**Background:**

The use of low-temperature plasma (LTP) is a novel approach to treating peri-implantitis. LTP disrupts the biofilm while conditioning the surrounding host environment for bone growth around the infected implant. The main objective of this study was to evaluate the antimicrobial properties of LTP on newly formed (24 h), intermediate (3 days), and mature (7 days) peri-implant-related biofilms formed on titanium surfaces.

**Methods:**

*Actinomyces naeslundii* (ATCC 12104), *Porphyromonas gingivalis* (W83), *Streptococcus oralis* (ATCC 35037), and *Veillonella dispar* (ATCC 17748) were cultivated in brain heart infusion supplemented with 1% yeast extract, hemin (0.5 mg/mL), and menadione (5 mg/mL) and kept at 37°C in anaerobic conditions for 24 h. Species were mixed for a final concentration of ~10^5^ colony forming units (CFU)/mL (OD = 0.01), and the bacterial suspension was put in contact with titanium specimens (7.5 mm in diameter by 2 mm in thickness) for biofilm formation. Biofilms were treated with LTP for 1, 3, and 5 min at 3 or 10 mm from plasma tip to sample. Controls were those having no treatment (negative control, NC) and argon flow under the same LTP conditions. Positive controls were those treated with 14 *μ*g/mL amoxicillin and 140 *μ*g/mL metronidazole individually or combined and 0.12% chlorhexidine (*n* = 6 per group). Biofilms were evaluated by CFU, confocal laser scanning microscopy (CLSM), and fluorescence in situ hybridization (FISH). Comparisons among bacteria; 24 h, 3-day, and 7-day biofilms; and treatments for each biofilm were made. Wilcoxon signed-rank and Wilcoxon rank sum tests were applied (*α* = 0.05).

**Results:**

Bacterial growth was observed in all NC groups, corroborated by FISH. LTP treatment significantly reduced all bacteria species compared to the NC in all biofilm periods and treatment conditions (*p* ≤ 0.016), and CLSM corroborated these results.

**Conclusion:**

Within the limitation of this study, we conclude that LTP application effectively reduces peri-implantitis-related multispecies biofilms on titanium surfaces *in vitro*.

## 1. Introduction

The oral cavity microbiota impact biofilm development on newly placed implants [1]. It is recognized that biofilm-induced inflammation around dental implants has a central role in stimulating osteoclast-mediated bone resorption and hindering bone formation, causing net bone loss around implants [1]. This process is known as peri-implantitis, a bacterium-started infection that yet has no effective treatment [2]. Healthy and systemically compromised patients can develop peri-implantitis, which may result in the loss of their dental implants [3]. The prevalence of peri-implantitis worldwide varies from 8.9% to 45% at the patient level and 4.8%–23.0% at the implant level [4, 5].

It has been verified that the biofilm around an infected implant presents a high occurrence of pathogens related to periodontitis [6], containing members of the red and orange complexes [7]. Common primary colonizers in the initial biofilm are streptococci, Veillonella, and Actinomyces [8–10]. *Streptococci* and *Actinomyces* species can coaggregate and offer attachment sites and growth support to other bacteria, such as *Veillonella* spp., that have metabolic interactions with *Streptococci* [11]. *Veillonella* species can also grow mixed communities with diverse late colonizers [12]. *Porphyromonas gingivalis*, known as a second colonizer [13], is often encountered at sites of peri-implantitis [14–16]. To evaluate antibiofilm therapies, proper models are required, for example, multispecies biofilm models, to simulate the *in vivo* condition [17].

Biofilm organization of the peri-implant bacteria prevents disease elimination by conventional antimicrobial treatment because of their poor penetration into the biofilm, along with adverse effects of the antimicrobial treatment associated with bacterial resistance and systemic toxicity. A few dentists appropriately prescribe antibiotics to their patients, which may result in some adverse effects, such as hypersensitivity and dermatological and allergic reactions, such as rashes, pruritus, and even anaphylactic shock [18]. Unnecessary prescription of antibiotics could result in numerous serious sequelae, for instance, gastric and hematological problems and alteration of resident bacterial microbita [18]. The overuse and misuse of penicillin and cephalosporins in dentistry have resulted in increased bacterial resistance triggered by the production of beta-lactamase. In addition, the risk of resistance might be augmented if penicillin is administered together with other antibiotics, for instance, metronidazole [18]. Current scientific evidence shows that in patients affected by peri-implantitis, the administration of systemic antibiotics did not reduce bleeding on probing or probing pocket depth [19]. Nevertheless, clinicians can expect significant results in reducing some secondary outcomes, such as reduced clinical attachment loss, lower suppuration and recession, reduced bone loss, and lower total bacterial counts. However, some adverse events, such as gastrointestinal effects, may also be triggered. It is suggested to avoid prescribing systemic antibiotics in the case of peri-implantitis to help manage the problem of antibiotic resistance [19].

Low-temperature plasma (LTP) has been documented as a powerful instrument for biomedicine and characterizes a new technology for treating peri-implantitis [20]. Unlike current peri-implantitis treatment strategies, preliminary data demonstrate LTP's unique potential to simultaneously decontaminate and/or detoxify biofilm-infected surfaces while creating a suitable environment for bone regrowth around the implant without damaging peri-implant healthy tissue [20–23]. LTP presents many advantages compared to, for example, the use of antibiotics, because it can be used for site-specific treatment; it provides (i) a quick bactericidal response; (ii) antimicrobial resistance is less likely to occur because of LTP's multiple modes of action and diversity of active agents [21], and (iii) there are minimal side effects [21]. Even though translational model evidence is necessary to establish the safety of LTP, tissue-compatible plasma temperatures encourage its *in vivo* use [22–23].

LTP produces high responsiveness at gas temperatures under 40°C. Electromagnetic radiation is produced, including ultraviolet radiation and light in the visible spectrum, including excited gas particles, charged ions, free electrons, free radicals, neutral reactive oxygen, and nitrogen species [24]. Oxygen and nitrogen-based radicals are considered the most significant contributors to LTP sterilizing effects [21]. The reactive species have robust oxidative effects on the external structures of bacterial cells. Unsaturated fats in the lipid bilayer of cell membranes are susceptible to attack by hydroxyl radicals, compromising membrane function, while proteins in membranes are susceptible to oxidative damage by reactive species. As soon as the cell membranes have been in part degraded, the reactive species may damage genetic material and molecules within the bacteria, ultimately destroying it [25]. Furthermore, the antimicrobial dosage of LTP exhibited no sign of relevant damage on a reconstituted oral epithelium [23] and reconstituted gingival epithelium [22], as demonstrated by low cytotoxicity and high cell viability levels [23]. In addition to the antimicrobial and tissue-friendly effects, multiple studies of LTP application on dental implant surfaces demonstrated that osseointegration increases up to 300% relative to nontreated implants resulting in superior implant biomechanical fixation in bone [26–28].

Given these advantages of LTP treatment, the present study is aimed at evaluating *in vitro* LTP's antimicrobial activity against 24 h, 3-day, and 7-day multispecies peri-implant-related biofilms formed by *Actinomyces naeslundii*, *P. gingivalis*, *Streptococcus oralis*, and *Veillonella dispar* at different exposure times (1, 3, or 5 min) and different distances from the equipment nozzle and the samples (3 or 10 mm). We hypothesize that a single LTP treatment *in vitro* will be sufficient to disrupt and disinfect multispecies peri-implantitis-related biofilm more effectively than the controls.

## 2. Material and Methods

### 2.1. Bacterial Strains, Growth Conditions, and Biofilm Formation

American Type Culture Collection (American Type Culture Collection (ATCC), Rockville, MD, USA) strains of *Actinomyces naeslundii* (ATCC 12104), *Porphyromonas gingivalis* (ATCC W83), *Streptococcus oralis* (ATCC 35037), and *Veillonella dispar* (ATCC 17748) were used to form the multispecies biofilms. Stock cultures were reactivated onto 5% sheep blood agar (Dickinson and Company; Sparks, MD, USA) plates and incubated at 37°C for 7 days. Bacteria were individually reactivated by transferring colonies to a tube containing 5 mL of brain heart infusion (BHI) broth culture medium (Acumedia; Lansing, MI, USA) supplemented with1% yeast extract, hemin (0.5 mg/mL), and menadione (5 mg/mL) and kept at 37°C in anaerobic conditions (80% N_2_, 10% H_2_, and 10% CO_2_) for 24 h. For all microorganisms, cocultures started from OD_600nm_ = 0.01 [17]. Four-species biofilm was developed on the surfaces of titanium discs made of commercially pure titanium grade 4 (Implacil De Bortoli; São Paulo, SP, Brazil). The discs' sizes are 7.5 mm in diameter by 2 mm in thickness, totaling an area of ~136 mm^2^. The microorganisms were mixed at the same ratio (1 : 1 : 1 : 1) at a final concentration of approximately 10^5^ CFU/mL (OD_600nm_ = 0.01), and 1 mL of the multispecies bacterial suspension was transferred to each well of a 24-well plate containing a titanium specimen. The plates were kept at 37°C in anaerobic conditions for 24 h, 3 days, and 7 days. For 3- and 7-day-old biofilms, the supplemented BHI broth was replaced daily.

### 2.2. Biofilm Treatments and Analysis

**Figure 1:**
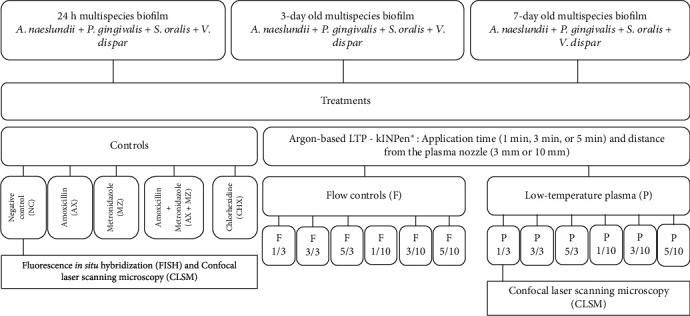
Flowchart representing the study design.

**Figure 2:**
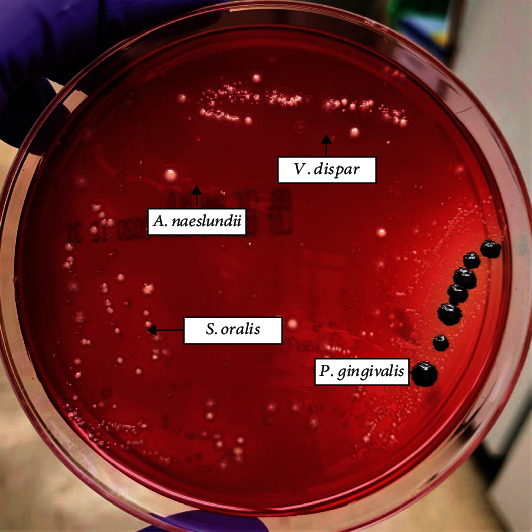
Four-species biofilm growth on blood agar plates. *A. naeslundii* shows crème/red-pigmented round colonies without hemolytic halo, *P. gingivalis* presents black-pigmented round colonies with a hemolysis halo, *S. oralis* grows characteristically in chains and forms small white colonies, and *V. dispar* colonies show a transparent halo with a white spot in the middle.

After each period, biofilms were treated with LTP (plasma—P) for 1 min, 3 min, and 5 min and 3 mm or 10 mm of distance between the plasma nozzle and the sample. Controls were those having no treatment (negative control—NC) and flow control (flow—F) under the same conditions (1, 3, or 5 min; 3 or 10 mm). In addition, 14 g/mL amoxicillin (AX) and 140 *μ*g/mL metronidazole (MZ) individually or in combination and 0.12% chlorhexidine (CHX) were used as positive controls (Figure 1). Biofilms formed on titanium discs were collected after treatments with a sterile spatula and transferred to tubes containing 5 mL of 0.89% NaCl. Biofilms' suspensions were sonicated (three 10 s pulses, 7 W output). An aliquot (0.1 mL) of the homogenized suspension was serially diluted and seeded onto 5% sheep blood agar blood plates. The plates were incubated at 37°C in anaerobic conditions for 7 days, and CFU/mL was calculated. Each bacterium was differentiated through its distinct morphology on the blood agar plates (Figure 2). The total of samples per group was 6 (*n* = 6). Steps 2.1 and 2.2 were performed at the Oral Microbiology laboratory at Indiana University School of Dentistry.

### 2.3. Fluorescence In Situ Hybridization (FISH)

FISH was performed according to Kommerein et al. [17] with few modifications. Biofilms from the NC of each period were rinsed once with Phosphate-Buffered Saline (PBS) (Thermo Fisher Scientific; Waltham, MA, USA) and fixed using a sequence of 50%, 80%, and 99% ethanol. This is a modification to Kommerein's [17] protocol to help with the dehydration of the samples, preventing dilution of the probe and hybridization buffer. After drying, the fixed cells in the biofilms were permeabilized with 1 *μ*g/*μ*L lysozyme for 15 min at 37°C. Lysis was stopped by adding absolute ethanol for 3 min; then, the samples were air-dried. Urea-NaCl buffer (pH 7.0) with 100 *μ*M of each probe was applied to the biofilms. The used probes were Alexa Fluor 488 for *A. naeslundii* (PMT detector; 488 nm laser), Alexa Fluor 647 for *P. gingivalis* (PMT detector; 638 nm laser), Alexa Fluor 405 for *S. oralis* (HyD detector; 405 nm laser), and Alexa Fluor 568 for *V. dispar* (HyD detector; 552 nm laser). Hybridization was made for 25 min at 46°C. Biofilms were rinsed twice with prewarmed urea-NaCl washing buffer (pH 7.0); then, the biofilms were incubated with this buffer for 5 min at 48°C. The washing steps were repeated twice. Biofilms were rinsed once with ultrapure water, PBS was added, and biofilms were visualized with a Leica SP8 DIVE confocal/2-photon microscope (Leica Microsystems, Buffalo Grove, IL, USA) and a Leica HC PL APO CS2 63x/1.3 Glyc objective lens (Leica Microsystems, Buffalo Grove, IL, USA). Images were acquired using a sequential illumination scanning mode with the following sequences: (1) Alexa Fluor 405 signals (HyD detector/405 nm laser/415-480 nm emission range) detected together with Alexa Fluor 568 signals (HyD detector/552 nm laser/560-630 nm emission range) and (2) Alexa Fluor 488 signals (PMT detector/488 nm laser/500-550 nm emission range) detected together with Alexa Fluor 647 (PMT detector/638 nm laser/650-750 nm emission range). Image stacks were acquired with an optimum z-step interval of 0.335 *μ*m.

### 2.4. Confocal Laser Scanning Microscopy (CLSM)

To demonstrate the effect of LTP treatment in a short period and distance of application, we performed CLSM illustrative pictures showing the structural organization of multispecies biofilms from the negative control and the treatment with LTP for 1 min at 3 mm in a 24 h, 3-day, and 7-day biofilms. Biofilms were stained with Live/Dead Bacterial Viability Kit (BacLight Bacterial Viability kit L7012; Thermo Fisher Scientific, Waltham, MA, USA) and incubated in the dark at room temperature for 15 minutes to permit infiltration of the fluorophores inside the bacterial cells. Specimens were rinsed twice with 0.89% NaCl and observed under a Leica SP8 Resonant-scanning confocal/multiphoton microscope (Leica Microsystems, Buffalo Grove, IL, USA) using Leica Fluotar VISIR 25x/0.95 water objective (Leica Microsystems, Buffalo Grove, IL, USA), with a free working distance of 2.3 mm. The pictures show live cells stained in green (Syto 9) while dead cells stained in red (Propidium Iodide). Steps 2.3 and 2.3 were performed at Indiana Center for Biological Microscopy (ICBM) at Indiana University School of Medicine.

### 2.5. Data Management and Statistical Analyses

Experiments were conducted in duplicate on three independent occasions (*n* = 6). The normal distribution of data was assessed by the Shapiro-Wilk test (*α* = 0.05). Data were not normally distributed, so nonparametric tests were used. Tests between bacteria were made using Wilcoxon signed rank tests, and tests between biofilms and between treatment groups were made using Wilcoxon rank sum tests. A 5% significance level was used. The analyses were done in the software SAS (SAS Institute, version 9.4, Cary, NC, USA). Comparisons were made among bacteria (6 comparisons among the 4 bacteria × 3 biofilms × 6 LTP − treated groups = 108); among 24 h, 3-day, and 7-day biofilms (3 biofilm comparisons × 4 bacteria × 6 LTP − treated groups = 72); and among treatments for each biofilm (6 LTP − treated groups × 6 control treatments [NC, AX, MZ, AX + MZ, CHX, flow control] + 9 comparisons among LTP − treated groups [3 within each plasma nozzle distance + 3 between plasma nozzle distances with same time]). The experiments were repeated three times, and the comparisons were made for the four species of bacteria, totaling 540 groups compared. Tables 1–5 show 95% confidence intervals (CI) and *p* values of group comparisons.

## 3. Results

### 3.1. Effect of LTP Treatments in *A. naeslundii* on the Multispecies Biofilm

In 24 h biofilms, all LTP-treated groups showed a significant reduction in *A. naeslundii* when compared to NC (*p* ≤ 0.014), AX (*p* ≤ 0.014), and flow controls (*p* ≤ 0.050). LTP and MZ treatments resulted in a similar reduction of *A. naeslundii* (*p* ≥ 0.240), which also happened between LTP and AX+MZ (*p* ≥ 0.088) and LTP and CHX (*p* ≥ 0.115).

In the 3-day-old biofilms, all LTP-treated groups significantly reduced *A. naeslundii* when compared to NC (*p* ≤ 0.017), AX (*p* ≤ 0.017), MZ (*p* ≤ 0.016), and AX+MZ (*p* ≤ 0.016). LTP in any tested conditions showed similar results as CHX for reducing *A. naeslundii* in the multispecies biofilm (*p* ≥ 0.051). LTP-treated groups showed a higher reduction of *A. naeslundii* than the flow controls (*p* ≤ 0.049), with the exception of LTP treatment for 1 minute at 10 mm (*p* = 0.170).

**Figure 3:**
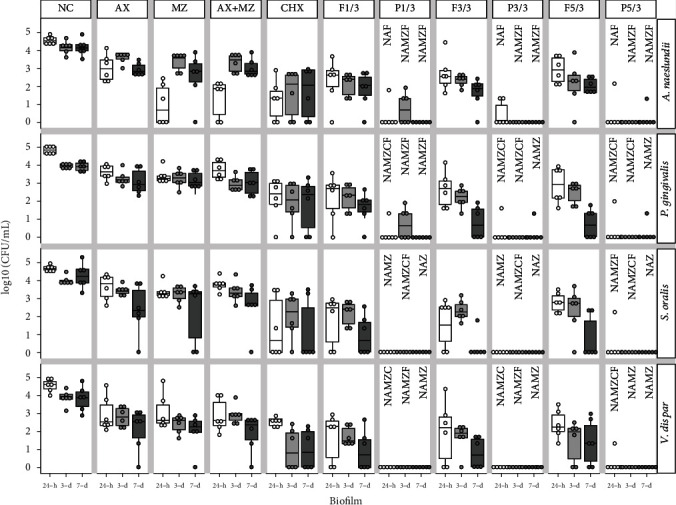
Box plot of log_10_ (CFU/mL) of 24 h, 3-day, and 7-day multispecies biofilms composed by *A. naeslundii*, *P. gingivalis*, *S. oralis*, and *V. dispar* at a distance of 3 mm from the equipment nozzle. NC stands for the negative control, which is the biofilms that received no treatment. P stands for LTP-treated groups for 1, 3, or 5 min (P1/3, P3/3, and P5/3), Flow (F) is the control for the LTP treatments (F1/3, F3/3, and F5/3). As positive controls, samples were treated with 0.12% chlorhexidine (CHX), amoxicillin (AX), metronizadole (MZ), and amoxicillin combined with metronidazole (AX+MZ). The letters represent statistical significance above each of the plasma treatments (*n* = 6; *p* ≤ 0.05; Wilcoxon signed rank and Wilcoxon rank sum tests). Letter *N* is for significance versus NC, letter *A* is for significance vs. AX, letter *M* is for significance vs. MZ, letter *Z* is for significance vs. AX+MZ, letter *C* is for significance vs. CHX, and letter *F* is for significance vs. its corresponding flow control. There were no statistically significant differences between the plasma treatments, between 24 h and 7 d within each plasma treatment, or between the bacteria within each plasma treatment.

**Figure 4:**
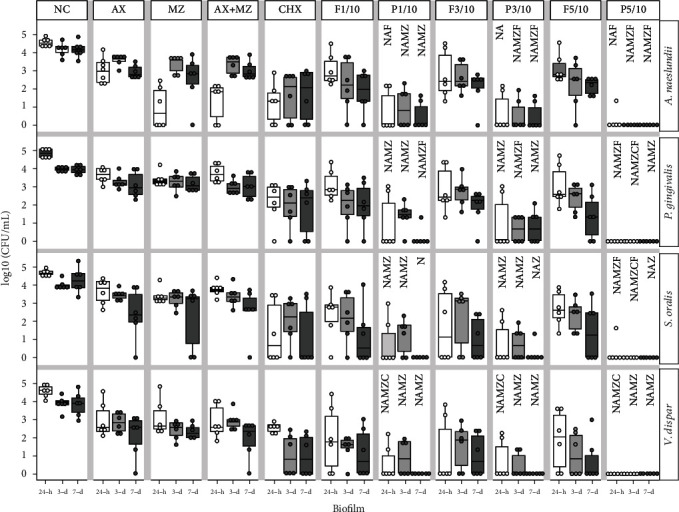
Box plot of log_10_ (CFU/mL) of 24 h, 3 d, and 7-day multispecies biofilms composed of *A. naeslundii*, *P. gingivalis*, *S. oralis*, and *V. dispar* at a distance of 10 mm from the equipment nozzle. NC stands for the negative control, which is the biofilms that received no treatment. P stands for LTP-treated groups for 1, 3, or 5 min (P1/10, P3/10, and P5/10), Flow (F) is the control for the LTP treatments (F1/10, F3/10, and /10). As positive controls, samples were treated with 0.12% chlorhexidine (CHX), amoxicillin (AX), metronizadole (MZ), and amoxicillin combined with metronidazole (AX+MZ). The letters represent statistical significance above each of the plasma treatments (*n* = 6; *p* ≤ 0.05; Wilcoxon signed rank and Wilcoxon rank sum tests). Letter *N* is for significance versus NC, letter *A* is for significance vs. AX, letter *M* is for significance vs. MZ, letter *Z* is for significance vs. AX+MZ, letter *C* is for significance vs. CHX, and letter *F* is for significance vs. its corresponding flow control. There were no statistically significant differences between the plasma treatments, between 24 h and 7 d within each plasma treatment, or between the bacteria within each plasma treatment.

In 7-day-old biofilms, there was also a significant reduction in *A. naeslundii* in all LTP-treated groups when compared to NC (*p* ≤ 0.016), AX (*p* ≤ 0.016), MZ (*p* ≤ 0.047), and AX+MZ (*p* ≤ 0.016). Similar outcomes were observed comparing LTP treatments with the positive control CHX (*p* ≥ 0.051). LTP treatment in all exposure times and distances reduced *A. naeslundii* in the biofilm compared to their respective flow controls (*p* ≤ 0.046), except for P1/10 (*p* = 0.107). See Figures 3 and 4.

### 3.2. Effect of LTP Treatments in *P. gingivalis* on the Multispecies Biofilm

In 24 h biofilms, LTP treatment at all exposure times and distances of application significantly reduced *P. gingivalis* when compared to NC (*p* ≤ 0.016). P1/3, P3/3, P5/3, and P5/10 significantly reduced *P. gingivalis* compared to CHX (*p* ≤ 0.048). All LTP treatments were more effective than AX (*p* ≤ 0.021), MZ (*p* ≤0.016), and AX+MZ (*p* ≤ 0.016).

In the 3-day-old biofilms, all LTP-treated groups significantly reduced *P. gingivalis* in comparison to the NC (*p* ≤ 0.017), AX (*p* ≤ 0.017), MZ (*p* ≤ 0.017), and AX+MZ (*p* ≤ 0.017). P1/3, P1/10, and P3/10 worked as efficiently as CHX (*p* ≥ 0.083). All LTP-treated groups showed a higher reduction of *P. gingivalis* than their flow controls (*p* ≤ 0.049), with the exception of P1/10 (*p* = 0.282).

In the 7-day-old biofilms, all LTP-treated groups significantly reduced *P. gingivalis* when compared to NC (*p* ≤ 0.016), AX (*p* ≤ 0.016), MZ (*p* ≤ 0.016), and AX+MZ (*p* ≤ 0.016). In contrast, LTP-treated groups showed similar results to CHX (*p* ≥ 0.051). Figures 3 and 4 show these results.

### 3.3. Effect of LTP Treatments in *S. oralis* on the Multispecies Biofilm

For the 24 h biofilm, all LTP treatments significantly reduced *S. oralis* when compared to NC (*p* ≤ 0.016), AX (*p* ≤ 0.021), MZ (*p* ≤ 0.016), and AX+MZ (*p* ≤ 0.016). Similar results in *S. oralis* reduction were observed for the LTP and CHX-treated groups (*p* ≥ 0.102). P5/3 and P5/10 significantly reduced *S. oralis* in comparison to their flow controls (*p* ≤ 0.022).

In the 3-day-old biofilms, LTP treatment at all exposure times and distances significantly reduced *S. oralis* when compared to the NC (*p* ≤ 0.017). P1/10 and P3/10 worked as efficiently as CHX (*p* ≥ 0.129). LTP-treated groups were significantly more effective in reducing *S. oralis* in the multispecies biofilm than the positive controls AX (*p* ≤ 0.017), MZ (*p* ≤ 0.017), and AX+MZ (*p* ≤ 0.017). All LTP-treated groups showed a higher reduction of *S. oralis* than their flow controls (*p* ≤ 0.025), with the exception of P1/10 (*p* = 0.223) and P3/10 (*p* = 0.185).

For 7-day-old biofilms, a significant reduction in *S. oralis* was observed for all the LTP-treated groups when compared to the NC (*p* ≤ 0.014). P1/3, P3/3, and P5/3 were more efficient in reducing *S. oralis* than AX (*p* ≤ 0.036) and AX+MZ (*p* ≤ 0.035). LTP-treated groups resulted in similar outcomes to the treatments with CHX (*p* ≥ 0.203) and MZ (*p* ≥ 0.051). LTP application results were all similar to their flow controls (*p* ≥ 0.102).

### 3.4. Effect of LTP Treatments in *V. dispar* on the Multispecies Biofilm

For 24 h biofilms, a significant reduction in *V. dispar* was observed in all LTP-treated groups when compared to the NC (*p* ≤ 0.016). Likewise, all LTP treatments were more efficient in reducing *V. dispar* in the multispecies biofilms than the positive controls CHX (*p* ≤ 0.018), AX (*p* ≤ 0.021), MZ (*p* ≤ 0.016), and AX+MZ (*p* ≤ 0.028). LTP application for 5 min at a distance of 3 mm (P5/3) significantly reduced *S. oralis* in the biofilm when compared to its flow control (F5/3) (*p* = 0.016).

For 3-day-old biofilms, all LTP treatments significantly reduced *V. dispar* compared to the NC (*p* ≤ 0.017). The use of LTP in all testing conditions showed similar results in the reduction of *V. dispar* abundance as observed in the CHX treatment group (*p* ≥ 0.102). All LTP treatments were more effective in reducing *V. dispar* in the multispecies biofilms than the other positive controls of AX (*p* ≤ 0.017), MZ (*p* ≤ 0.034), and AX+MZ (*p* ≤ 0.016). P1/3 and P3/3 showed a higher reduction of *V. dispar* when compared to their respective flow controls (*p* = 0.012 and *p* = 0.025, respectively), while the other LTP-treatments showed similar results to their flow controls (*p* ≥ 0.050).

For 7-day-old biofilms, LTP-treated groups significantly reduced V. dispar in comparison to the NC (*p* ≤ 0.012). All LTP treatments were also more effective in reducing *S. oralis* in the multispecies biofilms as compared to the positive controls of AX (*p* ≤ 0.025), MZ (*p* ≤ 0.025), and AX+MZ (*p* ≤ 0.025). In contrast, LTP-treated groups showed similar results in the reduction of *V. dispar* abundance as observed in the CHX-treated groups (*p* = 0.102). LTP application results were all similar to their respective flow controls (*p* ≥ 0.050) in the 7-day-old biofilms.

### 3.5. Comparison of LTP Treatments between 24 h, 3 Days, and 7 Days and among Each Bacterium

There were no statistically significant differences between 24 h, 3 days, and 7 days within each LTP treatment or among the bacteria within each LTP treatment (*p* ≥ 0.250).

### 3.6. Fluorescence In Situ Hybridization (FISH) and Confocal Laser Scanning Microscopy (CLSM)

**Figure 5:**
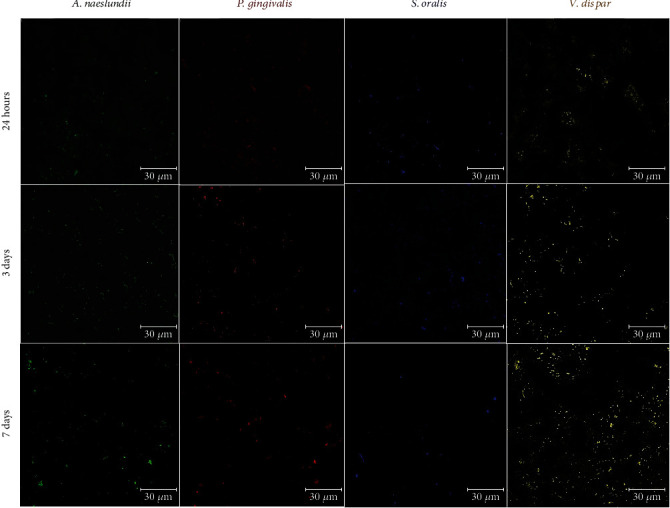
24 h, 3-day, and 7-day multispecies biofilms stained with FISH probes for *A. naeslundii* (Alexa Fluor-488; green), *P. gingivalis* (Alexa Fluor-647; red), *S. oralis* (Alexa Fluor-405; blue), and *V. dispar* (Alexa Fluor-568; yellow).

**Figure 6:**
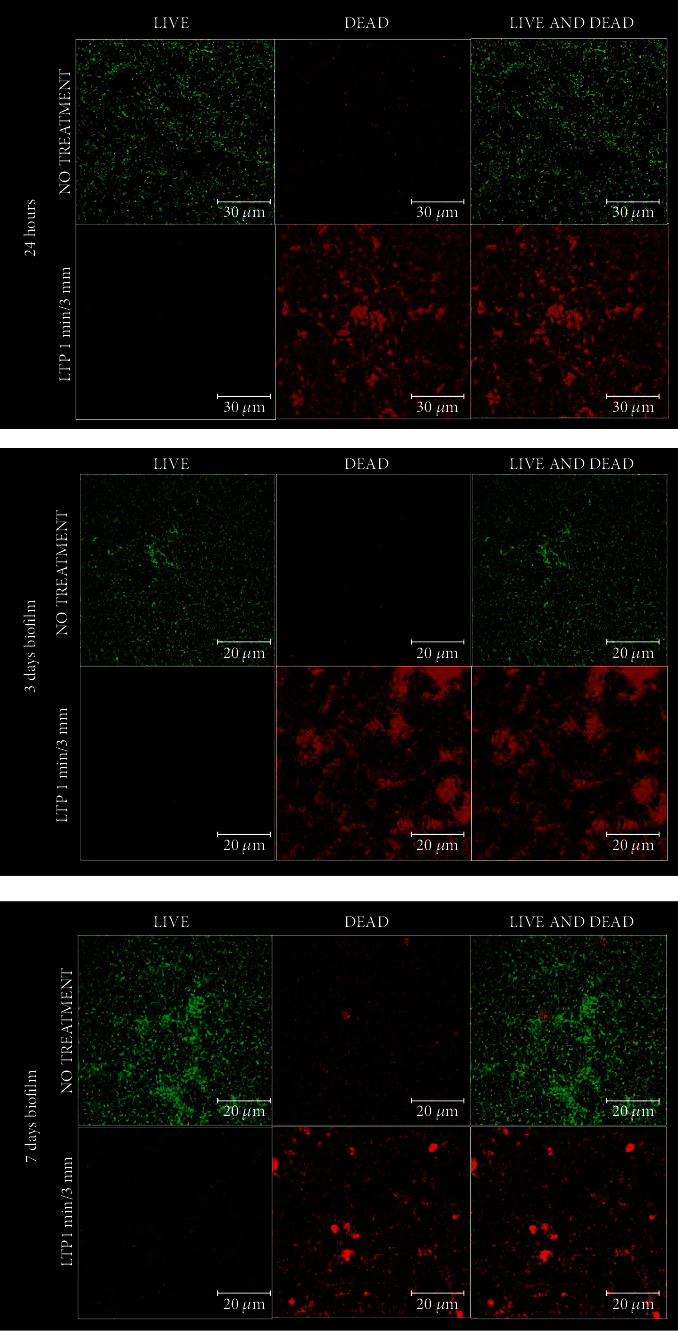
CLSM images of 24 h, 3-day, and 7-day multispecies biofilms. The images show live cells that are stained green (Syto 9) while dead cells are stained red (propidium iodine).

FISH images confirm the presence and the distribution of *A. naeslundii* (stained in green), *P. gingivalis* (stained in red), *S. oralis* (stained in blue), and *V. dispar* (stained in yellow) in the NC biofilms up to 7 days (Figure 5). To demonstrate the effect of LTP treatment in a short period and distance, Figure 6 depicts CLSM images showing the morphology and structural organization of multispecies biofilm before and after the treatment with LTP for 1 min at 3 mm (P1/3). The images confirm the CFU results obtained, showing a lower number of live cells (stained in green) in contrast to the increase in the number of dead cells (stained in red) in biofilms formed over 24 h, 3 days, and 7 days.

## 4. Discussion

Bacterial cells within a multispecies biofilm exhibit improved resistance to environmental surroundings, for instance, oxidative stress, antibiotic treatment, and nutrient depletion, when compared to planktonic cells. Furthermore, there is increasing evidence that multispecies relations in the biofilm extracellular matrix increase the resistance against disinfectants in comparison with single-species biofilms [29, 30]. In addition, the more mature the biofilm is, the more prone it is to have augmented resistance to antimicrobial therapies because of its antiphage action, protection against monoclonal antibodies, and protection exerted by components of the extracellular matrix [31]. Considering the importance of using a polymicrobial biofilm to simulate approach peri-implantitis conditions, we applied an *in vitro* biofilm model that was previously validated [17], composed of four important peri-implantitis-related bacteria. This variety of microorganisms in our biofilm model was confirmed using FISH probes, a molecular biology tool that we applied to detect the presence of *A. naeslundii*, *P. gingivalis*, *S. oralis*, and *V. dispar* in 24 h, 3-day, and 7-day-old biofilms.

Models to study biofilms *in vitro* gradually contribute to the existing data of biofilm physiology within the host condition and can be later translated into complex *in vivo* models of tissue infections. *In vitro* models have helped address elementary questions regarding biofilm development, physiology, and architecture. They offer many advantages such as a low cost, easy set-up, and responsiveness to high-throughput screens [32]. Therefore, in this study, we applied a validated model of *in vitro* peri-implantitis-related biofilm to test the effects of a single LTP treatment on different stages of biofilm formation.

The present study confirmed our hypothesis that a single LTP treatment *in vitro* was sufficient to disrupt and disinfect *in vitro* multispecies peri-implantitis-related biofilm more effectively than the controls. The application of LTP at 3 mm starting from 1 min demonstrated to be an effective measure in reducing the multispecies biofilm formed over 24 h, 3 days, and 7 days by *A. naeslundii*, *P. gingivalis*, *S. oralis*, and *V. dispar* on titanium surfaces. The application of LTP for 5 min demonstrated to be effective at both distances of 3 mm and 10 mm. Therefore, time of application and distance influenced the outcome of LTP treatments. With a shorter distance (3 mm) and the shortest period of application (1 min), LTP application was enough to reduce or kill bacteria in any of the tested periods of biofilm formation. With a longer distance (10 mm), the effectiveness of LTP depended on a longer period of application to obtain similar results as LTP 1 min at 3 mm. A previous study by Nicol et al. [21] showed a similar trend in results where the increase in distance between the jet nozzle and sample, as well as decreased voltage and flow rate, led to a decreased efficacy of LTP treatment. It is speculated that the addition of oxygen with longer distances between the sample and the LTP nozzle can lead to decreasing plume length, electron density, and ionization [33]. In contrast to time and distance of application, the age of the biofilms did not show to influence the LTP effects.

These results are remarkable since it is a short period of LTP application resulting in very few or zero bacterial recoveries. This was also confirmed through live and dead confocal images, which clearly showed that the treatment for 1 min at 3 mm with LTP killed bacteria on the titanium surfaces. It shows that LTP is penetrating and killing the bacteria within the biofilm. In contrast to current peri-implantitis treatment strategies, previous studies demonstrated that LTP has the unique potential to concurrently decontaminate and/or detoxify biofilm infected surfaces while creating a suitable environment for bone regrowth around the implant without harming peri-implant healthy host tissue [22–23, 26–27].

Antimicrobial resistance (AMR) is problematic at the therapeutic level in health sciences all over the world. According to the Global Burden of Bacterial Antimicrobial Resistance Report published in January 2022 [34], there were an estimated 4.95 million deaths associated with bacterial AMR in 2019, of which 1.27 million were attributable to bacterial AMR. The use of antimicrobials as assistants to periodontal mechanical therapy has been debatable. It is frequently kept for cases of aggravation of periodontal lesions or in patients who do not respond immediately to conventional mechanical therapy or patients who do not respond instantly to conventional mechanical therapy or in individuals who are in danger of having systemic compromise [35]. The combination of metronidazole-amoxicillin and metronidazole plus amoxicillin-clavulanate potassium appears to be the most effective antimicrobial therapy in the management of periodontal therapy [36]; however, a high number of periodontal disease-related bacteria that are resistant to these antibiotics have been reported [35]. In fact, we observed in our study that both metronidazole and amoxicillin were not effective in reducing the viability of the tested peri-implantitis-related biofilms *in vitro*. We believe this could have happened due to the presence of bulky biofilms. Biofilms' robust extracellular matrix and its components can block the access of bacterial biofilm communities from antibiotics and the host's immune cells [37]. Therefore, it is of high importance to look for possible treatments to avoid bacterial resistance; in this context, LTP is a great candidate as a coadjuvant or alternative therapy for peri-implantitis treatment since it will locally disrupt the biofilm. As LTP is a site-specific therapy, there is no systemic exposure, which means a lower chance of promoting antibiotic resistance. Moreover, LTP's multiple modes of action and diversity of active agents, such as reactive oxygen species, also diminish the likelihood of antibacterial resistance [21].

Tissue-compatible temperatures of plasma encourage LTP's use *in vivo* [22–23]. Recently, immediate and postrecovery cellular-level responses to LTP (helium-based) were examined *in vitro* using human gingival fibroblasts and osteoblasts (MC3T3-E1 cells) and *in vivo* in rat calvarial bone and the adjacent periosteum. The results showed that a direct LTP treatment for up to 3 min caused no harmful effects at either the cellular or tissue levels [38]. Dental implants previously treated with LTP showed greater bone-to-implant contact ratio, interthread, and peri-implant bone density [39]. Physicochemical and *in vivo* results demonstrated that air-based LTP treatment augmented surface energy and supported earlier osseointegration as compared to the controls [26]. LTP represents an innovative technology for the treatment of peri-implantitis. Clinically, LTP can potentially act as a coadjuvant to flap surgery in efficiently treating peri-implantitis, a necessary first step toward the development of novel efficient treatments to increase the longevity and success rate of millions of implants currently in function. LTP, coupled with surgical debridement, might be a unique efficacious approach for treating peri-implantitis by reducing biofilm viability, increasing the levels of peri-implant tissue reattachment, and enhancing implant surface characteristics for bone tissue healing. Moreover, LTP devices are sufficiently small for safe and portable operation in clinical settings and provide appropriate energy to cause meaningful alteration to biomaterial surfaces [20]. LTP antibiofilm activity against multispecies mature biofilm can go beyond the oral cavity, being a potential candidate as a safe antimicrobial coadjuvant for other types of implants, such as joint-replacement implants [40]. As a multipurpose and powerful tool, LTP is capable of upgrading surgical implants using various strategies of interface biotechnology, for instance, surface modification, coating deposition, and drug delivery [41], in addition to being antimicrobial, as we have shown in this study.

As a limitation, this is an *in vitro* study done in ideal laboratory conditions. It does not count the host's immune system, salivary proteins, the presence of a systemic disease, or other patient factors that would influence biofilm's response to LTP treatment. There are also implant surface aspects that need to be considered. When an implant is fixed in the human body, they are coated with blood proteins and interstitial fluids. This process is determined by the implant's surface chemistry and wettability. As soon as implanted, bacteria use adhesins to attach to the implant surface. It is suggested that factors such as surface roughness, free energy, chemistry, and titanium purity, besides the patient's periodontal condition, might impact the microbiome [42]. Therefore, future studies using an *in vivo* model should be performed, and implant surface characteristic evaluation regarding bacterial attachment is encouraged. The present study established parameters that will be further tested in a more complex *in vivo* model.

Within the limitations of this study, we conclude that the LTP application is a promising treatment to disinfect the peri-implant surroundings, which can potentially act as a coadjuvant therapy during the surgical procedure. Furthermore, LTP is effective against biofilms at a short distance of 3 mm starting from a short exposure time of 1 min.

Tables 1–5 show the statistical analysis of group comparisons for log_10_ (CFU/mL). Number of samples (*n*) analyzed per group was 6. The 95% confidence intervals (CI) and the *p* values (*α* = 0.05) are presented in the tables.

## Figures and Tables

**Table 1:** Comparisons between bacteria.

Biofilm	Group	Comparison			*p* value	Result	95% CI
24 h	Plasma 1 min/3 mm	*A. naeslundii*	vs.	*P. gingivalis*	1.000	*A. naeslundii* & *P. gingivalis n.s.*	0.00 (-1.32, 1.79)
		*A. naeslundii*	vs.	*S. oralis*	1.000	*A. naeslundii* & *S. oralis n.s.*	0.00 (0.00, 1.79)
		*A. naeslundii*	vs.	*V. dispar*	1.000	*A. naeslundii* & *V. dispar n.s.*	0.00 (0.00, 1.79)
		*P. gingivalis*	vs.	*S. oralis*	1.000	*P. gingivalis* & *S. oralis n.s.*	0.00 (0.00, 1.32)
		*P. gingivalis*	vs.	*V. dispar*	1.000	*P. gingivalis* & *V. dispar n.s.*	0.00 (0.00, 1.32)
		*S. oralis*	vs.	*V. dispar*	1.000	*S. oralis* & *V. dispar n.s.*	—
	Plasma 1 min/10 mm	*A. naeslundii*	vs.	*P. gingivalis*	0.500	*A. naeslundii* & *P. gingivalis n.s.*	-0.31 (-0.83, 0.00)
		*A. naeslundii*	vs.	*S. oralis*	1.000	*A. naeslundii* & *S. oralis n.s.*	0.00 (-0.75, 0.36)
		*A. naeslundii*	vs.	*V. dispar*	1.000	*A. naeslundii* & *V. dispar n.s.*	0.00 (0.00, 0.83)
		*P. gingivalis*	vs.	*S. oralis*	0.500	*P. gingivalis* & *S. oralis n.s.*	0.04 (0.00, 0.99)
		*P. gingivalis*	vs.	*V. dispar*	0.500	*P. gingivalis* & *V. dispar n.s.*	0.41 (0.00, 1.46)
		*S. oralis*	vs.	*V. dispar*	0.500	*S. oralis* & *V. dispar n.s.*	0.23 (0.00, 0.75)
	Plasma 3 min/3 mm	*A. naeslundii*	vs.	*P. gingivalis*	1.000	*A. naeslundii* & *P. gingivalis n.s.*	0.00 (-1.61, 1.32)
		*A. naeslundii*	vs.	*S. oralis*	0.500	*A. naeslundii* & *S. oralis n.s.*	0.66 (0.00, 1.32)
		*A. naeslundii*	vs.	*V. dispar*	0.500	*A. naeslundii* & *V. dispar n.s.*	0.66 (0.00, 1.32)
		*P. gingivalis*	vs.	*S. oralis*	1.000	*P. gingivalis* & *S. oralis n.s.*	0.00 (0.00, 1.61)
		*P. gingivalis*	vs.	*V. dispar*	1.000	*P. gingivalis* & *V. dispar n.s.*	0.00 (0.00, 1.61)
		*S. oralis*	vs.	*V. dispar*	1.000	*S. oralis* & *V. dispar n.s.*	—
	Plasma 3 min/10 mm	*A. naeslundii*	vs.	*P. gingivalis*	0.500	*A. naeslundii* & *P. gingivalis n.s.*	-0.40 (-0.88, 0.00)
		*A. naeslundii*	vs.	*S. oralis*	0.500	*A. naeslundii* & *S. oralis n.s.*	-0.05 (-0.45, 0.00)
		*A. naeslundii*	vs.	*V. dispar*	0.500	*A. naeslundii* & *V. dispar n.s.*	-0.03 (-0.10, 0.00)
		*P. gingivalis*	vs.	*S. oralis*	0.500	*P. gingivalis* & *S. oralis n.s.*	0.22 (0.00, 0.71)
		*P. gingivalis*	vs.	*V. dispar*	0.500	*P. gingivalis* & *V. dispar n.s.*	0.36 (0.00, 0.83)
		*S. oralis*	vs.	*V. dispar*	1.000	*S. oralis* & *V. dispar n.s.*	0.00 (0.00, 0.40)
	Plasma 5 min/3 mm	*A. naeslundii*	vs.	*P. gingivalis*	1.000	*A. naeslundii* & *P. gingivalis n.s.*	0.00 (0.00, 0.14)
		*A. naeslundii*	vs.	*S. oralis*	1.000	*A. naeslundii* & *S. oralis n.s.*	0.00 (-0.06, 0.00)
		*A. naeslundii*	vs.	*V. dispar*	1.000	*A. naeslundii* & *V. dispar n.s.*	0.00 (0.00, 0.83)
		*P. gingivalis*	vs.	*S. oralis*	1.000	*P. gingivalis* & *S. oralis n.s.*	0.00 (-0.20, 0.00)
		*P. gingivalis*	vs.	*V. dispar*	1.000	*P. gingivalis* & *V. dispar n.s.*	0.00 (0.00, 0.68)
		*S. oralis*	vs.	*V. dispar*	1.000	*S. oralis* & *V. dispar n.s.*	0.00 (0.00, 0.88)
	Plasma 5 min/10 mm	*A. naeslundii*	vs.	*P. gingivalis*	1.000	*A. naeslundii* & *P. gingivalis n.s.*	0.00 (0.00, 1.32)
		*A. naeslundii*	vs.	*S. oralis*	1.000	*A. naeslundii* & *S. oralis n.s.*	0.00 (-0.29, 0.00)
		*A. naeslundii*	vs.	*V. dispar*	1.000	*A. naeslundii* & *V. dispar n.s.*	0.00 (0.00, 1.32)
		*P. gingivalis*	vs.	*S. oralis*	1.000	*P. gingivalis* & *S. oralis n.s.*	0.00 (-1.61, 0.00)
		*P. gingivalis*	vs.	*V. dispar*	1.000	*P. gingivalis* & *V. dispar n.s.*	—
		*S. oralis*	vs.	*V. dispar*	1.000	*S. oralis* & *V .dispar n.s.*	0.00 (0.00, 1.61)
3 d	Plasma 1 min/3 mm	*A. naeslundii*	vs.	*P. gingivalis*	1.000	*A. naeslundii* & *P. gingivalis n.s.*	0.00 (-1.32, 1.32)
		*A. naeslundii*	vs.	*S. oralis*	0.250	*A. naeslundii* & *S. oralis n.s.*	0.66 (0.00, 1.91)
		*A. naeslundii*	vs.	*V. dispar*	0.250	*A. naeslundii* & *V. dispar n.s.*	0.66 (0.00, 1.91)
		*P. gingivalis*	vs.	*S. oralis*	0.250	*P. gingivalis* & *S. oralis n.s.*	0.66 (0.00, 1.91)
		*P. gingivalis*	vs.	*V. dispar*	0.250	*P. gingivalis* & *V. dispar n.s.*	0.66 (0.00, 1.91)
		*S. oralis*	vs.	*V. dispar*	1.000	*S. oralis* & *V. dispar n.s.*	—
	Plasma 1 min/10 mm	*A. naeslundii*	vs.	*P. gingivalis*	0.500	*A. naeslundii* & *P. gingivalis n.s.*	-0.66 (-1.32, 0.00)
		*A. naeslundii*	vs.	*S. oralis*	1.000	*A. naeslundii* & *S. oralis n.s.*	0.00 (-1.32, 0.00)
		*A. naeslundii*	vs.	*V. dispar*	1.000	*A. naeslundii* & *V. dispar n.s.*	0.00 (0.00, 0.39)
		*P. gingivalis*	vs.	*S. oralis*	1.000	*P. gingivalis* & *S. oralis n.s.*	0.00 (0.00, 1.32)
		*P. gingivalis*	vs.	*V. dispar*	0.250	*P. gingivalis* & *V. dispar n.s.*	0.66 (0.00, 1.32)
		*S. oralis*	vs.	*V. dispar*	0.500	*S. oralis* & *V. dispar n.s.*	0.20 (0.00, 1.32)
	Plasma 3 min/3 mm	*A. naeslundii*	vs.	*P. gingivalis*	1.000	*A. naeslundii* & *P. gingivalis n.s.*	—
		*A. naeslundii*	vs.	*S. oralis*	1.000	*A. naeslundii* & *S. oralis n.s.*	—
		*A. naeslundii*	vs.	*V. dispar*	1.000	*A. naeslundii* & *V. dispar n.s.*	—
		*P. gingivalis*	vs.	*S. oralis*	1.000	*P. gingivalis* & *S. oralis n.s.*	—
		*P. gingivalis*	vs.	*V. dispar*	1.000	*P. gingivalis* & *V. dispar n.s.*	—
		*S. oralis*	vs.	*V. dispar*	1.000	*S. oralis* & *V. dispar n.s.*	—
	Plasma 3 min/10 mm	*A. naeslundii*	vs.	*P. gingivalis*	1.000	*A. naeslundii* & *P. gingivalis n.s.*	0.00 (-1.32, 0.59)
		*A. naeslundii*	vs.	*S. oralis*	1.000	*A. naeslundii* & *S. oralis n.s.*	0.00 (-1.32, 0.00)
		*A. naeslundii*	vs.	*V. dispar*	1.000	*A. naeslundii* & *V. dispar n.s.*	0.00 (0.00, 0.59)
		*P. gingivalis*	vs.	*S. oralis*	1.000	*P. gingivalis* & *S. oralis n.s.*	0.00 (-0.59, 0.00)
		*P. gingivalis*	vs.	*V. dispar*	1.000	*P. gingivalis* & *V. dispar n.s.*	0.00 (0.00, 1.32)
		*S. oralis*	vs.	*V. dispar*	0.500	*S. oralis* & *V. dispar n.s.*	0.29 (0.00, 1.32)
	Plasma 5 min/3 mm	*A. naeslundii*	vs.	*P. gingivalis*	1.000	*A. naeslundii* & *P. gingivalis n.s.*	—
		*A. naeslundii*	vs.	*S. oralis*	1.000	*A. naeslundii* & *S. oralis n.s.*	—
		*A. naeslundii*	vs.	*V. dispar*	1.000	*A. naeslundii* & *V. dispar n.s.*	—
		*P. gingivalis*	vs.	*S. oralis*	1.000	*P. gingivalis* & *S. oralis n.s.*	—
		*P. gingivalis*	vs.	*V. dispar*	1.000	*P. gingivalis* & *V. dispar n.s.*	—
		*S. oralis*	vs.	*V. dispar*	1.000	*S. oralis* & *V. dispar n.s.*	—
	Plasma 5 min/10 mm	*A. naeslundii*	vs.	*P. gingivalis*	1.000	*A. naeslundii* & *P. gingivalis n.s.*	—
		*A. naeslundii*	vs.	*S. oralis*	1.000	*A. naeslundii* & *S. oralis n.s.*	—
		*A. naeslundii*	vs.	*V. dispar*	1.000	*A. naeslundii* & *V. dispar n.s.*	—
		*P. gingivalis*	vs.	*S. oralis*	1.000	*P. gingivalis* & *S. oralis n.s.*	—
		*P. gingivalis*	vs.	*V. dispar*	1.000	*P. gingivalis* & *V. dispar n.s.*	—
		*S. oralis*	vs.	*V. dispar*	1.000	*S. oralis* & *V. dispar n.s.*	—
7 d	Plasma 1 min/3 mm	*A. naeslundii*	vs.	*P. gingivalis*	1.000	*A. naeslundii* & *P. gingivalis n.s.*	—
		*A. naeslundii*	vs.	*S. oralis*	1.000	*A. naeslundii* & *S. oralis n.s.*	—
		*A. naeslundii*	vs.	*V. dispar*	1.000	*A. naeslundii* & *V. dispar n.s.*	—
		*P. gingivalis*	vs.	*S. oralis*	1.000	*P. gingivalis & S. oralis n.s.*	—
		*P. gingivalis*	vs.	*V. dispar*	1.000	*P. gingivalis* & *V. dispar n.s.*	—
		*S. oralis*	vs.	*V. dispar*	1.000	*S. oralis* & *V. dispar n.s.*	—
	Plasma 1 min/10 mm	*A. naeslundii*	vs.	*P. gingivalis*	0.750	*A. naeslundii* & *P. gingivalis n.s.*	0.00 (-1.32, 1.61)
		*A. naeslundii*	vs.	*S. oralis*	1.000	*A. naeslundii* & *S. oralis n.s.*	0.00 (-2.75, 1.61)
		*A. naeslundii*	vs.	*V. dispar*	0.500	*A. naeslundii* & *V. dispar n.s.*	0.66 (0.00, 1.61)
		*P. gingivalis*	vs.	*S. oralis*	1.000	*P. gingivalis* & *S. oralis n.s.*	0.00 (-2.75, 1.32)
		*P. gingivalis*	vs.	*V. dispar*	1.000	*P. gingivalis* & *V. dispar n.s.*	0.00 (0.00, 1.32)
		*S. oralis*	vs.	*V. dispar*	1.000	*S. oralis* & *V. dispar n.s.*	0.00 (0.00, 2.75)
	Plasma 3 min/3 mm	*A. naeslundii*	vs.	*P. gingivalis*	1.000	*A. naeslundii* & *P. gingivalis n.s.*	0.00 (-1.32, 0.00)
		*A. naeslundii*	vs.	*S. oralis*	1.000	*A. naeslundii* & *S. oralis n.s.*	—
		*A. naeslundii*	vs.	*V. dispar*	1.000	*A. naeslundii* & *V. dispar n.s.*	—
		*P. gingivalis*	vs.	*S. oralis*	1.000	*P. gingivalis* & *S. oralis n.s.*	0.00 (0.00, 1.32)
		*P. gingivalis*	vs.	*V. dispar*	1.000	*P. gingivalis* & *V. dispar n.s.*	0.00 (0.00, 1.32)
		*S. oralis*	vs.	*V. dispar*	1.000	*S. oralis* & *V. dispar n.s.*	—
	Plasma 3 min/10 mm	*A. naeslundii*	vs.	*P. gingivalis*	0.813	*A. naeslundii* & *P. gingivalis n.s.*	-0.24 (-2.08, 1.61)
		*A. naeslundii*	vs.	*S. oralis*	0.750	*A. naeslundii* & *S. oralis n.s.*	0.00 (-1.32, 1.61)
		*A. naeslundii*	vs.	*V. dispar*	0.500	*A. naeslundii* & *V. dispar n.s.*	0.66 (0.00, 1.61)
		*P. gingivalis*	vs.	*S. oralis*	0.500	*P. gingivalis* & *S. oralis n.s.*	0.66 (-1.32, 2.08)
		*P. gingivalis*	vs.	*V. dispar*	0.250	*P. gingivalis* & *V. dispar n.s.*	0.66 (0.00, 2.08)
		*S. oralis*	vs.	*V. dispar*	1.000	*S. oralis* & *V. dispar n.s.*	0.00 (0.00, 1.32)
	Plasma 5 min/3 mm	*A. naeslundii*	vs.	*P. gingivalis*	1.000	*A. naeslundii* & *P. gingivalis n.s.*	—
		*A. naeslundii*	vs.	*S. oralis*	1.000	*A. naeslundii* & *S. oralis n.s.*	0.00 (0.00, 1.32)
		*A. naeslundii*	vs.	*V. dispar*	1.000	*A. naeslundii* & *V. dispar n.s.*	0.00 (0.00, 1.32)
		*P. gingivalis*	vs.	*S. oralis*	1.000	*P. gingivalis* & *S. oralis n.s.*	0.00 (0.00, 1.32)
		*P. gingivalis*	vs.	*V. dispar*	1.000	*P. gingivalis* & *V. dispar n.s.*	0.00 (0.00, 1.32)
		*S. oralis*	vs.	*V. dispar*	1.000	*S. oralis* & *V. dispar n.s.*	—
	Plasma 5 min/10 mm	*A. naeslundii*	vs.	*P. gingivalis*	1.000	*A. naeslundii* & *P. gingivalis n.s.*	—
		*A. naeslundii*	vs.	*S. oralis*	1.000	*A. naeslundii* & *S. oralis n.s.*	—
		*A. naeslundii*	vs.	*V. dispar*	1.000	*A. naeslundii* & *V. dispar n.s.*	—
		*P. gingivalis*	vs.	*S. oralis*	1.000	*P. gingivalis* & *S. oralis n.s.*	—
		*P. gingivalis*	vs.	*V. dispar*	1.000	*P. gingivalis* & *V. dispar n.s.*	—
		*S. oralis*	vs.	*V. dispar*	1.000	*S. oralis* & *V. dispar n.s.*	—

**Table 2:** Comparisons between biofilms.

Bacteria	Biofilms	Group	*p* value	Result	95% CI
*A. naeslundii*	24 h vs. 3 d	Plasma 1 min/3 mm	0.360	24 h & 3 d n.s.	0.00 (-1.32, 0.00)
		Plasma 1 min/10 mm	0.794	24 h & 3 d n.s.	0.00 (-1.79, 0.59)
		Plasma 3 min/3 mm	0.201	24 h & 3 d n.s.	0.00 (0.00, 1.32)
		Plasma 3 min/10 mm	0.852	24 h & 3 d n.s.	0.00 (-1.32, 1.91)
		Plasma 5 min/3 mm	0.422	24 h & 3 d n.s.	—
		Plasma 5 min/10 mm	0.422	24 h & 3 d n.s.	—
	24 h vs. 7 d	Plasma 1 min/3 mm	0.422	24 h & 7 d n.s.	—
		Plasma 1 min/10 mm	0.780	24 h & 7 d n.s.	0.00 (-1.32, 2.15)
		Plasma 3 min/3 mm	0.201	24 h & 7 d n.s.	0.00 (0.00, 1.32)
		Plasma 3 min/10 mm	0.780	24 h & 7 d n.s.	0.00 (-1.32, 1.91)
		Plasma 5 min/3 mm	1.000	24 h & 7 d n.s.	—
		Plasma 5 min/10 mm	0.422	24 h & 7 d n.s.	—
	3 d vs. 7 d	Plasma 1 min/3 mm	0.101	3 d & 7 d n.s.	0.66 (0.00, 1.32)
		Plasma 1 min/10 mm	0.390	3 d & 7 d n.s.	0.00 (0.00, 1.79)
		Plasma 3 min/3 mm	1.000	3 d & 7 d n.s.	—
		Plasma 3 min/10 mm	1.000	3 d & 7 d n.s.	0.00 (-1.32, 1.32)
		Plasma 5 min/3 mm	0.422	3 d & 7 d n.s.	—
		Plasma 5 min/10 mm	1.000	3 d & 7 d n.s.	—
*P. gingivalis*	24 h vs. 3 d	Plasma 1 min/3 mm	0.273	24 h & 3 d n.s.	0.00 (-1.32, 0.00)
		Plasma 1 min/10 mm	0.572	24 h & 3 d n.s.	-1.32 (-1.79, 1.46)
		Plasma 3 min/3 mm	0.422	24 h & 3 d n.s.	—
		Plasma 3 min/10 mm	1.000	24 h & 3 d n.s.	0.00 (-1.32, 1.71)
		Plasma 5 min/3 mm	0.422	24 h & 3 d n.s.	—
		Plasma 5 min/10 mm	1.000	24 h & 3 d n.s.	—
	24 h vs. 7 d	Plasma 1 min/3 mm	0.422	24 h & 7 d n.s.	—
		Plasma 1 min/10 mm	0.477	24 h & 7 d n.s.	0.00 (0.00, 2.78)
		Plasma 3 min/3 mm	1.000	24 h & 7 d n.s.	—
		Plasma 3 min/10 mm	1.000	24 h & 7 d n.s.	0.00 (-1.32, 1.71)
		Plasma 5 min/3 mm	1.000	24 h & 7 d n.s.	—
		Plasma 5 min/10 mm	1.000	24 h & 7 d n.s.	—
	3 d vs. 7 d	Plasma 1 min/3 mm	0.101	3 d & 7 d n.s.	0.66 (0.00, 1.32)
		Plasma 1 min/10 mm	0.047	3 d > 7 d	1.32 (0.00, 1.79)
		Plasma 3 min/3 mm	0.422	3 d & 7 d n.s.	—
		Plasma 3 min/10 mm	0.862	3 d & 7 d n.s.	0.00 (-1.32, 1.32)
		Plasma 5 min/3 mm	0.422	3 d & 7 d n.s.	—
		Plasma 5 min/10 mm	1.000	3 d & 7 d n.s.	—
*S. oralis*	24 h vs. 3 d	Plasma 1 min/3 mm	1.000	24 h & 3 d n.s.	—
		Plasma 1 min/10 mm	0.618	24 h & 3 d n.s.	0.00 (-1.79, 1.34)
		Plasma 3 min/3 mm	1.000	24 h & 3 d n.s.	—
		Plasma 3 min/10 mm	1.000	24 h & 3 d n.s.	0.00 (-1.32, 1.28)
		Plasma 5 min/3 mm	0.422	24 h & 3 d n.s.	—
		Plasma 5 min/10 mm	0.422	24 h & 3 d n.s.	—
	24 h vs. 7 d	Plasma 1 min/3 mm	1.000	24 h & 7 d n.s.	—
		Plasma 1 min/10 mm	0.610	24 h & 7 d n.s.	0.00 (0.00, 1.79)
		Plasma 3 min/3 mm	1.000	24 h & 7 d n.s.	—
		Plasma 3 min/10 mm	0.477	24 h & 7 d n.s.	0.00 (0.00, 2.00)
		Plasma 5 min/3 mm	0.422	24 h & 7 d n.s.	—
		Plasma 5 min/10 mm	0.422	24 h & 7 d n.s.	—
	3 d vs. 7 d	Plasma 1 min/3 mm	1.000	3 d & 7 d n.s.	—
		Plasma 1 min/10 mm	0.270	3 d & 7 d n.s.	1.32 (0.00, 1.79)
		Plasma 3 min/3 mm	1.000	3 d & 7 d n.s.	—
		Plasma 3 min/10 mm	0.273	3 d & 7 d n.s.	0.00 (0.00, 1.32)
		Plasma 5 min/3 mm	1.000	3 d & 7 d n.s.	—
		Plasma 5 min/10 mm	1.000	3 d & 7 d n.s.	—
*V. dispar*	24 h vs. 3 d	Plasma 1 min/3 mm	1.000	24 h & 3 d n.s.	—
		Plasma 1 min/10 mm	0.664	24 h & 3 d n.s.	0.00 (-1.79, 0.59)
		Plasma 3 min/3 mm	1.000	24 h & 3 d n.s.	—
		Plasma 3 min/10 mm	0.780	24 h & 3 d n.s.	0.00 (-1.32, 2.00)
		Plasma 5 min/3 mm	0.422	24 h & 3 d n.s.	—
		Plasma 5 min/10 mm	1.000	24 h & 3 d n.s.	—
	24 h vs. 7 d	Plasma 1 min/3 mm	1.000	24 h & 7 d n.s.	—
		Plasma 1 min/10 mm	0.203	24 h & 7 d n.s.	0.00 (0.00, 1.32)
		Plasma 3 min/3 mm	1.000	24 h & 7 d n.s.	—
		Plasma 3 min/10 mm	0.203	24 h & 7 d n.s.	0.00 (0.00, 2.00)
		Plasma 5 min/3 mm	0.422	24 h & 7 d n.s.	—
		Plasma 5 min/10 mm	1.000	24 h & 7 d n.s.	—
	3 d vs. 7 d	Plasma 1 min/3 mm	1.000	3 d & 7 d n.s.	—
		Plasma 1 min/10 mm	0.102	3 d & 7 d n.s.	0.81 (0.00, 1.79)
		Plasma 3 min/3 mm	1.000	3 d & 7 d n.s.	—
		Plasma 3 min/10 mm	0.201	3 d & 7 d n.s.	0.00 (0.00, 1.32)
		Plasma 5 min/3 mm	1.000	3 d & 7 d n.s.	—
		Plasma 5 min/10 mm	1.000	3 d & 7 d n.s.	—

**Table 3:** Comparisons between LPT groups.

Bacteria	Biofilm	Comparison		Group 2	*p* value	Result	95% CI
*A. naeslundii*	24 h	Plasma 1 min/3 mm	vs.	Plasma 1 min/10 mm	0.477	Plasma 1 min/3 mm & plasma 1 min/10 mm n.s.	0.00 (-2.15, 0.00)
		Plasma 1 min/3 mm	vs.	Plasma 3 min/3 mm	0.758	plasma 1 min/3 mm & plasma 3 min/3 mm n.s.	0.00 (-1.32, 0.00)
		Plasma 1 min/3 mm	vs.	Plasma 5 min/3 mm	1.000	Plasma 1 min/3 mm & plasma 5 min/3 mm n.s.	—
		Plasma 1 min/10 mm	vs.	Plasma 3 min/10 mm	0.852	Plasma 1 min/10 mm & plasma 3 min/10 mm n.s.	0.00 (-1.91, 2.15)
		Plasma 1 min/10 mm	vs.	Plasma 5 min/10 mm	0.477	Plasma 1 min/10 mm & plasma 5 min/10 mm n.s.	0.00 (0.00, 2.15)
		Plasma 3 min/3 mm	vs.	Plasma 3 min/10 mm	0.780	Plasma 3 min/3 mm & plasma 3 min/10 mm n.s.	0.00 (-1.91, 1.32)
		Plasma 3 min/3 mm	vs.	Plasma 5 min/3 mm	0.758	Plasma 3 min/3 mm & plasma 5 min/3 mm n.s.	0.00 (0.00, 1.32)
		Plasma 3 min/10 mm	vs.	Plasma 5 min/10 mm	0.477	Plasma 3 min/10 mm & plasma 5 min/10 mm n.s.	0.00 (0.00, 1.91)
		Plasma 5 min/3 mm	vs.	Plasma 5 min/10 mm	1.000	Plasma 5 min/3 mm & plasma 5 min/10 mm n.s.	—
	3 d	Plasma 1 min/3 mm	vs.	Plasma 1 min/10 mm	0.738	Plasma 1 min/3 mm & plasma 1 min/10 mm n.	0.00 (-1.79, 1.32)
		Plasma 1 min/3 mm	vs.	Plasma 3 min/3 mm	0.101	Plasma 1 min/3 mm & plasma 3 min/3 mm n.s.	0.66 (0.00, 1.32)
		Plasma 1 min/3 mm	vs.	Plasma 5 min/3 mm	0.101	Plasma 1 min/3 mm & plasma 5 min/3 mm n.s.	0.66 (0.00, 1.32)
		Plasma 1 min/10 mm	vs.	Plasma 3 min/10 mm	0.545	Plasma 1 min/10 mm & plasma 3 min/10 mm	0.00 (-0.30, 1.79)
		Plasma 1 min/10 mm	vs.	Plasma 5 min/10 mm	0.102	Plasma 1 min/10 mm & plasma 5 min/10 mm	0.81 (0.00, 1.79)
		Plasma 3 min/3 mm	vs.	Plasma 3 min/10 mm	0.203	Plasma 3 min/3 mm & plasma 3 min/10 mm n.	0.00 (-1.32, 0.00)
		Plasma 3 min/3 mm	vs.	Plasma 5 min/3 mm	1.000	Plasma 3 min/3 mm & plasma 5 min/3 mm n.s.	—
		Plasma 3 min/10 mm	vs.	Plasma 5 min/10 mm	0.203	Plasma 3 min/10 mm & plasma 5 min/10 mm	0.00 (0.00, 1.32)
		Plasma 5 min/3 mm	vs.	Plasma 5 min/10 mm	1.000	Plasma 5 min/3 mm & plasma 5 min/10 mm n.	—
	7 d	Plasma 1 min/3 mm	vs.	Plasma 1 min/10 mm	0.203	Plasma 1 min/3 mm & plasma 1 min/10 mm n.s.	0.00 (-1.32, 0.00)
		Plasma 1 min/3 mm	vs.	Plasma 3 min/3 mm	1.000	Plasma 1 min/3 mm & plasma 3 min/3 mm n.s.	—
		Plasma 1 min/3 mm	vs.	Plasma 5 min/3 mm	0.422	Plasma 1 min/3 mm & plasma 5 min/3 mm n.s.	—
		Plasma 1 min/10 mm	vs.	Plasma 3 min/10 mm	1.000	Plasma 1 min/10 mm & plasma 3 min/10 mm n.s.	0.00 (-1.32, 1.32)
		Plasma 1 min/10 mm	vs.	Plasma 5 min/10 mm	0.203	Plasma 1 min/10 mm & plasma 5 min/10 mm n.s.	0.00 (0.00, 1.32)
		Plasma 3 min/3 mm	vs.	Plasma 3 min/10 mm	0.203	Plasma 3 min/3 mm & plasma 3 min/10 mm n.s.	0.00 (-1.32, 0.00)
		Plasma 3 min/3 mm	vs.	Plasma 5 min/3 mm	0.422	Plasma 3 min/3 mm & plasma 5 min/3 mm n.s.	—
		Plasma 3 min/10 mm	vs.	Plasma 5 min/10 mm	0.203	Plasma 3 min/10 mm & plasma 5 min/10 mm n.s.	0.00 (0.00, 1.32)
		Plasma 5 min/3 mm	vs.	Plasma 5 min/10 mm	0.422	Plasma 5 min/3 mm & plasma 5 min/10 mm n.s.	—
*P. gingivalis*	24 h	Plasma 1 min/3 mm	vs.	Plasma 1 min/10 mm	0.477	Plasma 1 min/3 mm & plasma 1 min/10 mm n.s.	0.00 (-2.78, 0.00)
		Plasma 1 min/3 mm	vs.	Plasma 3 min/3 mm	1.000	Plasma 1 min/3 mm & plasma 3 min/3 mm n.s.	—
		Plasma 1 min/3 mm	vs.	Plasma 5 min/3 mm	1.000	Plasma 1 min/3 mm & plasma 5 min/3 mm n.s.	—
		Plasma 1 min/10 mm	vs.	Plasma 3 min/10 mm	1.000	Plasma 1 min/10 mm & plasma 3 min/10 mm n.s.	0.00 (-2.72, 2.78)
		Plasma 1 min/10 mm	vs.	Plasma 5 min/10 mm	0.203	Plasma 1 min/10 mm & plasma 5 min/10 mm n.s.	0.00 (0.00, 2.78)
		Plasma 3 min/3 mm	vs.	Plasma 3 min/10 mm	0.477	Plasma 3 min/3 mm & plasma 3 min/10 mm n.s.	0.00 (-2.72, 0.00)
		Plasma 3 min/3 mm	vs.	Plasma 5 min/3 mm	1.000	Plasma 3 min/3 mm & plasma 5 min/3 mm n.s.	—
		Plasma 3 min/10 mm	vs.	Plasma 5 min/10 mm	0.203	Plasma 3 min/10 mm & plasma 5 min/10 mm n.s.	0.00 (0.00, 2.72)
		Plasma 5 min/3 mm	vs.	Plasma 5 min/10 mm	0.422	Plasma 5 min/3 mm & plasma 5 min/10 mm n.s.	—
	3 d	Plasma 1 min/3 mm	vs.	Plasma 1 min/10 mm	0.270	Plasma 1 min/3 mm & plasma 1 min/10 mm n.	-0.46 (-1.79, 0.30)
		Plasma 1 min/3 mm	vs.	Plasma 3 min/3 mm	0.101	Plasma 1 min/3 mm & plasma 3 min/3 mm n.s.	0.66 (0.00, 1.32)
		Plasma 1 min/3 mm	vs.	Plasma 5 min/3 mm	0.101	Plasma 1 min/3 mm & plasma 5 min/3 mm n.s.	0.66 (0.00, 1.32)
		Plasma 1 min/10 mm	vs.	Plasma 3 min/10 mm	0.119	Plasma 1 min/10 mm & plasma 3 min/10 mm	0.72 (0.00, 1.79)
		Plasma 1 min/10 mm	vs.	Plasma 5 min/10 mm	0.025	Plasma 1 min/10 mm > plasma 5 min/10 mm	1.47 (1.32, 1.79)
		Plasma 3 min/3 mm	vs.	Plasma 3 min/10 mm	0.098	plasma 3 min/3 mm & plasma 3 min/10 mm n.	-0.66 (-1.32, 0.00)
		Plasma 3 min/3 mm	vs.	Plasma 5 min/3 mm	1.000	Plasma 3 min/3 mm & plasma 5 min/3 mm n.s.	—
		Plasma 3 min/10 mm	vs.	Plasma 5 min/10 mm	0.098	Plasma 3 min/10 mm & plasma 5 min/10 mm	0.66 (0.00, 1.32)
		Plasma 5 min/3 mm	vs.	Plasma 5 min/10 mm	1.000	Plasma 5 min/3 mm & plasma 5 min/10 mm n.	—
	7 d	Plasma 1 min/3 mm	vs.	Plasma 1 min/10 mm	0.422	Plasma 1 min/3 mm & plasma 1 min/10 mm n.s.	—
		Plasma 1 min/3 mm	vs.	Plasma 3 min/3 mm	0.422	Plasma 1 min/3 mm & plasma 3 min/3 mm n.s.	—
		Plasma 1 min/3 mm	vs.	Plasma 5 min/3 mm	0.422	Plasma 1 min/3 mm & plasma 5 min/3 mm n.s.	—
		Plasma 1 min/10 mm	vs.	Plasma 3 min/10 mm	0.273	Plasma 1 min/10 mm & plasma 3 min/10 mm n.s.	0.00 (-1.32, 0.00)
		Plasma 1 min/10 mm	vs.	Plasma 5 min/10 mm	0.422	Plasma 1 min/10 mm & plasma 5 min/10 mm n.s.	—
		Plasma 3 min/3 mm	vs.	Plasma 3 min/10 mm	0.273	Plasma 3 min/3 mm & plasma 3 min/10 mm n.s.	0.00 (-1.32, 0.00)
		Plasma 3 min/3 mm	vs.	Plasma 5 min/3 mm	1.000	Plasma 3 min/3 mm & plasma 5 min/3 mm n.s.	—
		Plasma 3 min/10 mm	vs.	Plasma 5 min/10 mm	0.101	Plasma 3 min/10 mm & plasma 5 min/10 mm n.s.	0.66 (0.00, 1.32)
		Plasma 5 min/3 mm	vs.	Plasma 5 min/10 mm	0.422	Plasma 5 min/3 mm & plasma 5 min/10 mm n.s.	—
*S. oralis*	24 h	Plasma 1 min/3 mm	vs.	Plasma 1 min/10 mm	0.203	Plasma 1 min/3 mm & plasma 1 min/10 mm n.s.	0.00 (-1.79, 0.00)
		Plasma 1 min/3 mm	vs.	Plasma 3 min/3 mm	1.000	Plasma 1 min/3 mm & plasma 3 min/3 mm n.s.	—
		Plasma 1 min/3 mm	vs.	Plasma 5 min/3 mm	0.422	Plasma 1 min/3 mm & plasma 5 min/3 mm n.s.	—
		Plasma 1 min/10 mm	vs.	Plasma 3 min/10 mm	1.000	Plasma 1 min/10 mm & plasma 3 min/10 mm n.s.	0.00 (-2.00, 1.79)
		Plasma 1 min/10 mm	vs.	Plasma 5 min/10 mm	0.477	Plasma 1 min/10 mm & plasma 5 min/10 mm n.s.	0.00 (0.00, 1.79)
		Plasma 3 min/3 mm	vs.	Plasma 3 min/10 mm	0.203	Plasma 3 min/3 mm & plasma 3 min/10 mm n.s.	0.00 (-2.00, 0.00)
		Plasma 3 min/3 mm	vs.	Plasma 5 min/3 mm	0.422	Plasma 3 min/3 mm & plasma 5 min/3 mm n.s.	—
		Plasma 3 min/10 mm	vs.	Plasma 5 min/10 mm	0.477	Plasma 3 min/10 mm & plasma 5 min/10 mm n.s.	0.00 (0.00, 2.00)
		Plasma 5 min/3 mm	vs.	Plasma 5 min/10 mm	1.000	Plasma 5 min/3 mm & plasma 5 min/10 mm n.s.	—
	3 d	Plasma 1 min/3 mm	vs.	Plasma 1 min/10 mm	0.051	Plasma 1 min/3 mm & plasma 1 min/10 mm n.	-1.47 (-1.79, 0.00)
		Plasma 1 min/3 mm	vs.	Plasma 3 min/3 mm	1.000	Plasma 1 min/3 mm & plasma 3 min/3 mm n.s.	—
		Plasma 1 min/3 mm	vs.	Plasma 5 min/3 mm	1.000	Plasma 1 min/3 mm & plasma 5 min/3 mm n.s.	—
		Plasma 1 min/10 mm	vs.	Plasma 3 min/10 mm	0.467	Plasma 1 min/10 mm & plasma 3 min/10 mm	0.29 (-1.32, 1.79)
		Plasma 1 min/10 mm	vs.	Plasma 5 min/10 mm	0.051	Plasma 1 min/10 mm & plasma 5 min/10 mm	1.47 (0.00, 1.79)
		Plasma 3 min/3 mm	vs.	Plasma 3 min/10 mm	0.101	Plasma 3 min/3 mm & plasma 3 min/10 mm n.	-0.66 (-1.32, 0.00)
		Plasma 3 min/3 mm	vs.	Plasma 5 min/3 mm	1.000	Plasma 3 min/3 mm & plasma 5 min/3 mm n.s.	—
		Plasma 3 min/10 mm	vs.	Plasma 5 min/10 mm	0.101	Plasma 3 min/10 mm & plasma 5 min/10 mm	0.66 (0.00, 1.32)
		Plasma 5 min/3 mm	vs.	Plasma 5 min/10 mm	1.000	Plasma 5 min/3 mm & plasma 5 min/10 mm n.	—
	7 d	Plasma 1 min/3 mm	vs.	Plasma 1 min/10 mm	0.422	Plasma 1 min/3 mm & plasma 1 min/10 mm n.s.	—
		Plasma 1 min/3 mm	vs.	Plasma 3 min/3 mm	1.000	Plasma 1 min/3 mm & plasma 3 min/3 mm n.s.	—
		Plasma 1 min/3 mm	vs.	Plasma 5 min/3 mm	1.000	Plasma 1 min/3 mm & plasma 5 min/3 mm n.s.	—
		Plasma 1 min/10 mm	vs.	Plasma 3 min/10 mm	1.000	Plasma 1 min/10 mm & plasma 3 min/10 mm n.s.	—
		Plasma 1 min/10 mm	vs.	Plasma 5 min/10 mm	0.422	Plasma 1 min/10 mm & plasma 5 min/10 mm n.s.	—
		Plasma 3 min/3 mm	vs.	Plasma 3 min/10 mm	0.422	Plasma 3 min/3 mm & plasma 3 min/10 mm n.s.	—
		Plasma 3 min/3 mm	vs.	Plasma 5 min/3 mm	1.000	Plasma 3 min/3 mm & plasma 5 min/3 mm n.s.	—
		Plasma 3 min/10 mm	vs.	Plasma 5 min/10 mm	0.422	Plasma 3 min/10 mm & plasma 5 min/10 mm n.s.	—
		Plasma 5 min/3 mm	vs.	Plasma 5 min/10 mm	1.000	Plasma 5 min/3 mm & plasma 5 min/10 mm n.s.	—
*V. dispar*	24 h	Plasma 1 min/3 mm	vs.	Plasma 1 min/10 mm	0.203	Plasma 1 min/3 mm & plasma 1 min/10 mm n.s.	0.00 (-1.32, 0.00)
		Plasma 1 min/3 mm	vs.	Plasma 3 min/3 mm	1.000	Plasma 1 min/3 mm & plasma 3 min/3 mm n.s.	—
		Plasma 1 min/3 mm	vs.	Plasma 5 min/3 mm	0.422	Plasma 1 min/3 mm & plasma 5 min/3 mm n.s.	—
		Plasma 1 min/10 mm	vs.	Plasma 3 min/10 mm	1.000	Plasma 1 min/10 mm & plasma 3 min/10 mm n.s.	0.00 (-2.00, 1.32)
		Plasma 1 min/10 mm	vs.	Plasma 5 min/10 mm	0.203	Plasma 1 min/10 mm & plasma 5 min/10 mm n.s.	0.00 (0.00, 1.32)
		Plasma 3 min/3 mm	vs.	Plasma 3 min/10 mm	0.203	Plasma 3 min/3 mm & plasma 3 min/10 mm n.s.	0.00 (-2.00, 0.00)
		Plasma 3 min/3 mm	vs.	Plasma 5 min/3 mm	0.422	Plasma 3 min/3 mm & plasma 5 min/3 mm n.s.	—
		Plasma 3 min/10 mm	vs.	Plasma 5 min/10 mm	0.203	Plasma 3 min/10 mm & plasma 5 min/10 mm n.s.	0.00 (0.00, 2.00)
		Plasma 5 min/3 mm	vs.	Plasma 5 min/10 mm	0.422	Plasma 5 min/3 mm & plasma 5 min/10 mm n.s.	—
	3 d	Plasma 1 min/3 mm	vs.	Plasma 1 min/10 mm	0.102	Plasma 1 min/3 mm & plasma 1 min/10 mm n.	-0.81 (-1.79, 0.00)
		Plasma 1 min/3 mm	vs.	Plasma 3 min/3 mm	1.000	Plasma 1 min/3 mm & plasma 3 min/3 mm n.s.	—
		Plasma 1 min/3 mm	vs.	Plasma 5 min/3 mm	1.000	Plasma 1 min/3 mm & plasma 5 min/3 mm n.s.	—
		Plasma 1 min/10 mm	vs.	Plasma 3 min/10 mm	0.346	Plasma 1 min/10 mm & plasma 3 min/10 mm	0.15 (0.00, 1.79)
		Plasma 1 min/10 mm	vs.	Plasma 5 min/10 mm	0.102	Plasma 1 min/10 mm & plasma 5 min/10 mm	0.81 (0.00, 1.79)
		Plasma 3 min/3 mm	vs.	Plasma 3 min/10 mm	0.201	Plasma 3 min/3 mm & plasma 3 min/10 mm n.	0.00 (-1.32, 0.00)
		Plasma 3 min/3 mm	vs.	Plasma 5 min/3 mm	1.000	Plasma 3 min/3 mm & plasma 5 min/3 mm n.s.	—
		Plasma 3 min/10 mm	vs.	Plasma 5 min/10 mm	0.201	Plasma 3 min/10 mm & plasma 5 min/10 mm	0.00 (0.00, 1.32)
		Plasma 5 min/3 mm	vs.	Plasma 5 min/10 mm	1.000	Plasma 5 min/3 mm & plasma 5 min/10 mm n.	—
	7 d	Plasma 1 min/3 mm	vs.	Plasma 1 min/10 mm	1.000	Plasma 1 min/3 mm & plasma 1 min/10 mm n.s.	—
		Plasma 1 min/3 mm	vs.	Plasma 3 min/3 mm	1.000	Plasma 1 min/3 mm & plasma 3 min/3 mm n.s.	—
		Plasma 1 min/3 mm	vs.	Plasma 5 min/3 mm	1.000	Plasma 1 min/3 mm & plasma 5 min/3 mm n.s.	—
		Plasma 1 min/10 mm	vs.	Plasma 3 min/10 mm	1.000	Plasma 1 min/10 mm & plasma 3 min/10 mm n.s.	—
		Plasma 1 min/10 mm	vs.	Plasma 5 min/10 mm	1.000	Plasma 1 min/10 mm & plasma 5 min/10 mm n.s.	—
		Plasma 3 min/3 mm	vs.	Plasma 3 min/10 mm	1.000	Plasma 3 min/3 mm & plasma 3 min/10 mm n.s.	—
		Plasma 3 min/3 mm	vs.	Plasma 5 min/3 mm	1.000	Plasma 3 min/3 mm & plasma 5 min/3 mm n.s.	—
		Plasma 3 min/10 mm	vs.	Plasma 5 min/10 mm	1.000	Plasma 3 min/10 mm & plasma 5 min/10 mm n.s.	—
		Plasma 5 min/3 mm	vs.	Plasma 5 min/10 mm	1.000	Plasma 5 min/3 mm & plasma 5 min/10 mm n.s.	—

**Table 4:** Comparisons LPT groups against flow control.

Bacteria	Biofilm	Comparison			*p* value	Result	95% CI
*A. naeslundii*	24 h	Plasma 1 min/3 mm	vs.	Flow 1 min/3 mm	0.041	Plasma 1 min/3 mm < flow 1 min/3 mm	-2.62 (-2.92, 0.00)
		Plasma 1 min/10 mm	vs.	Flow 1 min/10 mm	0.016	Plasma 1 min/10 mm < flow 1 min/10 mm	-2.48 (-3.70, -0.33)
		Plasma 3 min/3 mm	vs.	Flow 3 min/3 mm	0.016	Plasma 3 min/3 mm < flow 3 min/3 mm	-2.28 (-3.12, -1.16)
		Plasma 3 min/10 mm	vs.	Flow 3 min/10 mm	0.050	Plasma 3 min/10 mm & flow 3 min/10 mm n.s.	-2.26 (-4.30, -0.11)
		Plasma 5 min/3 mm	vs.	Flow 5 min/3 mm	0.022	Plasma 5 min/3 mm < flow 5 min/3 mm	-2.60 (-3.70, -1.57)
		Plasma 5 min/10 mm	vs.	Flow 5 min/10 mm	0.014	Plasma 5 min/10 mm < flow 5 min/10 mm	-2.76 (-3.62, -2.45)
	3 d	Plasma 1 min/3 mm	vs.	Flow 1 min/3 mm	0.049	Plasma 1 min/3 mm < flow 1 min/3 mm	-1.32 (-2.56, 0.00)
		Plasma 1 min/10 mm	vs.	Flow 1 min/10 mm	0.170	Plasma 1 min/10 mm & flow 1 min/10 mm n.	-1.38 (-3.75, 0.46)
		Plasma 3 min/3 mm	vs.	Flow 3 min/3 mm	0.012	Plasma 3 min/3 mm < flow 3 min/3 mm	-2.43 (-2.64, -1.79)
		Plasma 3 min/10 mm	vs.	Flow 3 min/10 mm	0.021	Plasma 3 min/10 mm < flow 3 min/10 mm	-2.18 (-3.60, -0.67)
		Plasma 5 min/3 mm	vs.	Flow 5 min/3 mm	0.025	Plasma 5 min/3 mm < flow 5 min/3 mm	-2.30 (-2.70, -1.61)
		Plasma 5 min/10 mm	vs.	Flow 5 min/10 mm	0.025	Plasma 5 min/10 mm < flow 5 min/10 mm	-2.53 (-3.30, -1.32)
	7 d	Plasma 1 min/3 mm	vs.	Flow 1 min/3 mm	0.025	Plasma 1 min/3 mm < flow 1 min/3 mm	-2.00 (-2.64, -1.32)
		Plasma 1 min/10 mm	vs.	Flow 1 min/10 mm	0.107	Plasma 1 min/10 mm & flow 1 min/10 mm n.s.	-1.32 (-2.79, 0.00)
		Plasma 3 min/3 mm	vs.	Flow 3 min/3 mm	0.025	Plasma 3 min/3 mm < flow 3 min/3 mm	-1.89 (-2.15, -1.32)
		Plasma 3 min/10 mm	vs.	Flow 3 min/10 mm	0.046	Plasma 3 min/10 mm < flow 3 min/10 mm	-1.91 (-2.58, 0.00)
		Plasma 5 min/3 mm	vs.	Flow 5 min/3 mm	0.014	Plasma 5 min/3 mm < flow 5 min/3 mm	-1.79 (-2.48, -1.26)
		Plasma 5 min/10 mm	vs.	Flow 5 min/10 mm	0.012	Plasma 5 min/10 mm < flow 5 min/10 mm	-2.33 (-2.62, -1.61)
P. gingivalis	24 h	Plasma 1 min/3 mm	vs.	Flow 1 min/3 mm	0.041	Plasma 1 min/3 mm < flow 1 min/3 mm	-2.62 (-2.92, 0.00)
		Plasma 1 min/10 mm	vs.	Flow 1 min/10 mm	0.115	Plasma 1 min/10 mm & flow 1 min/10 mm n.s.	-2.48 (-3.81, 0.27)
		Plasma 3 min/3 mm	vs.	Flow 3 min/3 mm	0.019	Plasma 3 min/3 mm < flow 3 min/3 mm	-2.48 (-3.22, -1.61)
		Plasma 3 min/10 mm	vs.	Flow 3 min/10 mm	0.149	Plasma 3 min/10 mm & flow 3 min/10 mm n.s.	-2.26 (-4.29, 0.53)
		Plasma 5 min/3 mm	vs.	Flow 5 min/3 mm	0.019	Plasma 5 min/3 mm < flow 5 min/3 mm	-2.26 (-3.79, -1.61)
		Plasma 5 min/10 mm	vs.	Flow 5 min/10 mm	0.012	Plasma 5 min/10 mm < flow 5 min/10 mm	-2.57 (-4.72, -1.79)
	3 d	Plasma 1 min/3 mm	vs.	Flow 1 min/3 mm	0.049	Plasma 1 min/3 mm < flow 1 min/3 mm	-1.32 (-2.89, 0.00)
		Plasma 1 min/10 mm	vs.	Flow 1 min/10 mm	0.282	Plasma 1 min/10 mm & flow 1 min/10 mm n.	-0.70 (-1.80, 1.32)
		Plasma 3 min/3 mm	vs.	Flow 3 min/3 mm	0.012	Plasma 3 min/3 mm < flow 3 min/3 mm	-2.27 (-2.89, -1.32)
		Plasma 3 min/10 mm	vs.	Flow 3 min/10 mm	0.016	Plasma 3 min/10 mm < flow 3 min/10 mm	-1.93 (-3.03, -0.83)
		Plasma 5 min/3 mm	vs.	Flow 5 min/3 mm	0.012	Plasma 5 min/3 mm < flow 5 min/3 mm	-2.71 (-3.01, -1.61)
		Plasma 5 min/10 mm	vs.	Flow 5 min/10 mm	0.012	Plasma 5 min/10 mm < flow 5 min/10 mm	-2.62 (-3.20, -1.32)
	7 d	Plasma 1 min/3 mm	vs.	Flow 1 min/3 mm	0.025	Plasma 1 min/3 mm < flow 1 min/3 mm	-1.81 (-2.08, -1.32)
		Plasma 1 min/10 mm	vs.	Flow 1 min/10 mm	0.041	Plasma 1 min/10 mm < flow 1 min/10 mm	-1.79 (-3.20, 0.00)
		Plasma 3 min/3 mm	vs.	Flow 3 min/3 mm	0.240	Plasma 3 min/3 mm & flow 3 min/3 mm n.s.	0.00 (-1.61, 0.00)
		Plasma 3 min/10 mm	vs.	Flow 3 min/10 mm	0.076	Plasma 3 min/10 mm & flow 3 min/10 mm n.s.	-1.21 (-2.53, 0.00)
		Plasma 5 min/3 mm	vs.	Flow 5 min/3 mm	0.273	Plasma 5 min/3 mm & flow 5 min/3 mm n.s.	0.00 (-1.32, 0.00)
		Plasma 5 min/10 mm	vs.	Flow 5 min/10 mm	0.050	Plasma 5 min/10 mm & flow 5 min/10 mm n.s.	-1.32 (-2.45, 0.00)
*S. oralis*	24 h	Plasma 1 min/3 mm	vs.	Flow 1 min/3 mm	0.051	Plasma 1 min/3 mm & flow 1 min/3 mm n.s.	-2.46 (-2.75, 0.00)
		Plasma 1 min/10 mm	vs.	Flow 1 min/10 mm	0.162	Plasma 1 min/10 mm & flow 1 min/10 mm n.s.	-1.79 (-2.90, 0.05)
		Plasma 3 min/3 mm	vs.	Flow 3 min/3 mm	0.051	Plasma 3 min/3 mm & flow 3 min/3 mm n.s.	-2.43 (-2.56, 0.00)
		Plasma 3 min/10 mm	vs.	Flow 3 min/10 mm	0.439	Plasma 3 min/10 mm & flow 3 min/10 mm n.s.	0.00 (-3.82, 0.10)
		Plasma 5 min/3 mm	vs.	Flow 5 min/3 mm	0.022	Plasma 5 min/3 mm < flow 5 min/3 mm	-2.75 (-3.27, -1.30)
		Plasma 5 min/10 mm	vs.	Flow 5 min/10 mm	0.019	Plasma 5 min/10 mm < flow 5 min/10 mm	-2.45 (-3.68, -1.32)
	3 d	Plasma 1 min/3 mm	vs.	Flow 1 min/3 mm	0.012	Plasma 1 min/3 mm < flow 1 min/3 mm	-2.38 (-2.87, -1.32)
		Plasma 1 min/10 mm	vs.	Flow 1 min/10 mm	0.223	Plasma 1 min/10 mm & flow 1 min/10 mm n.	-1.21 (-2.48, 0.98)
		Plasma 3 min/3 mm	vs.	Flow 3 min/3 mm	0.012	Plasma 3 min/3 mm < flow 3 min/3 mm	-2.21 (-3.16, -1.61)
		Plasma 3 min/10 mm	vs.	Flow 3 min/10 mm	0.185	Plasma 3 min/10 mm & flow 3 min/10 mm n.	-1.78 (-3.27, 1.32)
		Plasma 5 min/3 mm	vs.	Flow 5 min/3 mm	0.025	Plasma 5 min/3 mm < flow 5 min/3 mm	-2.71 (-3.10, -1.79)
		Plasma 5 min/10 mm	vs.	Flow 5 min/10 mm	0.012	Plasma 5 min/10 mm < flow 5 min/10 mm	-2.53 (-3.44, -1.32)
	7 d	Plasma 1 min/3 mm	vs.	Flow 1 min/3 mm	0.102	Plasma 1 min/3 mm & flow 1 min/3 mm n.s.	-0.66 (-1.79, 0.00)
		Plasma 1 min/10 mm	vs.	Flow 1 min/10 mm	0.361	Plasma 1 min/10 mm & flow 1 min/10 mm n.s.	0.00 (-1.79, 0.00)
		Plasma 3 min/3 mm	vs.	Flow 3 min/3 mm	0.422	Plasma 3 min/3 mm & flow 3 min/3 mm n.s.	—
		Plasma 3 min/10 mm	vs.	Flow 3 min/10 mm	0.240	Plasma 3 min/10 mm & flow 3 min/10 mm n.s.	0.00 (-2.30, 0.00)
		Plasma 5 min/3 mm	vs.	Flow 5 min/3 mm	0.203	Plasma 5 min/3 mm & flow 5 min/3 mm n.s.	0.00 (-2.30, 0.00)
		Plasma 5 min/10 mm	vs.	Flow 5 min/10 mm	0.102	Plasma 5 min/10 mm & flow 5 min/10 mm n.s.	-1.21 (-2.45, 0.00)
*V. dispar*	24 h	Plasma 1 min/3 mm	vs.	Flow 1 min/3 mm	0.051	Plasma 1 min/3 mm & flow 1 min/3 mm n.s.	-2.26 (-2.62, 0.00)
		Plasma 1 min/10 mm	vs.	Flow 1 min/10 mm	0.226	Plasma 1 min/10 mm & flow 1 min/10 mm n.s.	-1.61 (-3.60, 0.30)
		Plasma 3 min/3 mm	vs.	Flow 3 min/3 mm	0.051	Plasma 3 min/3 mm & flow 3 min/3 mm n.s.	-2.19 (-2.90, 0.00)
		Plasma 3 min/10 mm	vs.	Flow 3 min/10 mm	0.780	Plasma 3 min/10 mm & flow 3 min/10 mm n.s.	0.00 (-3.26, 2.00)
		Plasma 5 min/3 mm	vs.	Flow 5 min/3 mm	0.016	Plasma 5 min/3 mm < flow 5 min/3 mm	-2.15 (-3.08, -1.32)
		Plasma 5 min/10 mm	vs.	Flow 5 min/10 mm	0.051	Plasma 5 min/10 mm & flow 5 min/10 mm n.s.	-2.00 (-3.53, 0.00)
	3 d	Plasma 1 min/3 mm	vs.	Flow 1 min/3 mm	0.012	Plasma 1 min/3 mm < flow 1 min/3 mm	-1.47 (-2.45, -1.32)
		Plasma 1 min/10 mm	vs.	Flow 1 min/10 mm	0.522	Plasma 1 min/10 mm & flow 1 min/10 mm n.	-0.12 (-1.91, 0.30)
		Plasma 3 min/3 mm	vs.	Flow 3 min/3 mm	0.025	Plasma 3 min/3 mm < flow 3 min/3 mm	-1.89 (-2.21, -1.61)
		Plasma 3 min/10 mm	vs.	Flow 3 min/10 mm	0.132	Plasma 3 min/10 mm & flow 3 min/10 mm n.	-1.32 (-2.38, 0.00)
		Plasma 5 min/3 mm	vs.	Flow 5 min/3 mm	0.050	Plasma 5 min/3 mm & flow 5 min/3 mm n.s.	-1.97 (-2.15, 0.00)
		Plasma 5 min/10 mm	vs.	Flow 5 min/10 mm	0.102	Plasma 5 min/10 mm & Flow 5 min/10 mm n.	-0.81 (-2.26, 0.00)
	7 d	Plasma 1 min/3 mm	vs.	Flow 1 min/3 mm	0.102	Plasma 1 min/3 mm & flow 1 min/3 mm n.s.	-0.66 (-1.61, 0.00)
		Plasma 1 min/10 mm	vs.	Flow 1 min/10 mm	0.102	Plasma 1 min/10 mm & flow 1 min/10 mm n.s.	-0.66 (-2.48, 0.00)
		Plasma 3 min/3 mm	vs.	Flow 3 min/3 mm	0.102	Plasma 3 min/3 mm & flow 3 min/3 mm n.s.	-0.66 (-1.61, 0.00)
		Plasma 3 min/10 mm	vs.	Flow 3 min/10 mm	0.102	Plasma 3 min/10 mm & flow 3 min/10 mm n.s.	-0.66 (-2.30, 0.00)
		Plasma 5 min/3 mm	vs.	Flow 5 min/3 mm	0.050	Plasma 5 min/3 mm & flow 5 min/3 mm n.s.	-1.32 (-2.66, 0.00)
		Plasma 5 min/10 mm	vs.	Flow 5 min/10 mm	0.203	Plasma 5 min/10 mm & flow 5 min/10 mm n.s.	0.00 (-1.32, 0.00)

**Table 5:** Comparisons against LPT groups.

Bacteria	Biofilm	Comparison		Group 2	*p* value	Result	95% CI
*A. naeslundii*	24 h	No treatment	vs.	Plasma 1 min/3 mm	0.014	No treatment > plasma 1 min/3 mm	4.38 (3.12, 4.70)
		No treatment	vs.	Plasma 1 min/10 mm	0.015	No treatment > plasma 1 min/10 mm	4.38 (2.23, 4.70)
		No treatment	vs.	Plasma 3 min/3 mm	0.015	No treatment > plasma 3 min/3 mm	4.38 (3.06, 4.70)
		No treatment	vs.	Plasma 3 min/10 mm	0.015	No treatment > plasma 3 min/10 mm	4.38 (2.47, 4.70)
		No treatment	vs.	Plasma 5 min/3 mm	0.014	No treatment > plasma 5 min/3 mm	4.38 (2.75, 4.70)
		No treatment	vs.	Plasma 5 min/10 mm	0.014	No treatment > plasma 5 min/10 mm	4.38 (3.58, 4.70)
		Chlorhexidine	vs.	Plasma 1 min/3 mm	0.156	Chlorhexidine & plasma 1 min/3 mm n.s.	1.32 (0.00, 1.91)
		Chlorhexidine	vs.	Plasma 1 min/10 mm	0.561	Chlorhexidine & plasma 1 min/10 mm n.s.	0.35 (-0.88, 1.91)
		Chlorhexidine	vs.	Plasma 3 min/3 mm	0.217	Chlorhexidine & plasma 3 min/3 mm n.s.	0.95 (0.00, 1.91)
		Chlorhexidine	vs.	Plasma 3 min/10 mm	0.507	Chlorhexidine & plasma 3 min/10 mm n.s.	0.38 (-0.83, 1.91)
		Chlorhexidine	vs.	Plasma 5 min/3 mm	0.207	Chlorhexidine & plasma 5 min/3 mm n.s.	1.32 (0.00, 1.91)
		Chlorhexidine	vs.	Plasma 5 min/10 mm	0.115	Chlorhexidine & plasma 5 min/10 mm n.s.	1.32 (0.00, 1.91)
		Amoxicillin	vs.	Plasma 1 min/3 mm	0.014	Amoxicillin > plasma 1 min/3 mm	2.60 (2.30, 3.53)
		Amoxicillin	vs.	Plasma 1 min/10 mm	0.016	Amoxicillin > plasma 1 min/10 mm	2.30 (0.40, 3.53)
		Amoxicillin	vs.	Plasma 3 min/3 mm	0.015	Amoxicillin > plasma 3 min/3 mm	2.45 (1.28, 3.53)
		Amoxicillin	vs.	Plasma 3 min/10 mm	0.016	Amoxicillin > plasma 3 min/10 mm	2.30 (0.45, 3.53)
		Amoxicillin	vs.	Plasma 5 min/3 mm	0.014	Amoxicillin > plasma 5 min/3 mm	2.60 (2.00, 3.53)
		Amoxicillin	vs.	Plasma 5 min/10 mm	0.014	Amoxicillin > plasma 5 min/10 mm	2.60 (2.30, 3.53)
		Metronidazole	vs.	Plasma 1 min/3 mm	0.277	Metronidazole & plasma 1 min/3 mm n.s.	0.00 (0.00, 2.08)
		Metronidazole	vs.	Plasma 1 min/10 mm	0.794	Metronidazole & plasma 1 min/10 mm n.s.	0.00 (-0.88, 2.08)
		Metronidazole	vs.	Plasma 3 min/3 mm	0.435	Metronidazole & plasma 3 min/3 mm n.s.	0.00 (0.00, 2.08)
		Metronidazole	vs.	Plasma 3 min/10 mm	0.664	Metronidazole & plasma 3 min/10 mm n.s.	0.00 (-0.83, 2.08)
		Metronidazole	vs.	Plasma 5 min/3 mm	0.361	Metronidazole & plasma 5 min/3 mm n.s.	0.00 (0.00, 2.08)
		Metronidazole	vs.	Plasma 5 min/10 mm	0.240	Metronidazole & plasma 5 min/10 mm n.s.	0.00 (0.00, 2.08)
		Amoxicillin+metronidazole	vs.	Plasma 1 min/3 mm	0.101	Amoxicillin+metronidazole & plasma 1 min/3 mm n.s.	1.79 (0.00, 2.08)
		Amoxicillin+metronidazole	vs.	Plasma 1 min/10 mm	0.618	Amoxicillin+metronidazole & Plasma 1 min/10 mm n.s.	0.00 (-0.36, 2.08)
		Amoxicillin+metronidazole	vs.	Plasma 3 min/3 mm	0.132	Amoxicillin+metronidazole & plasma 3 min/3 mm n.s.	0.82 (0.00, 2.08)
		Amoxicillin+metronidazole	vs.	Plasma 3 min/10 mm	0.410	Amoxicillin+metronidazole & plasma 3 min/10 mm n.s.	0.12 (-0.24, 2.08)
		Amoxicillin+metronidazole	vs.	Plasma 5 min/3 mm	0.208	Amoxicillin+metronidazole & plasma 5 min/3 mm n.s.	1.79 (0.00, 2.08)
		Amoxicillin+metronidazole	vs.	Plasma 5 min/10 mm	0.088	Amoxicillin+metronidazole & plasma 5 min/10 mm n.s.	1.79 (0.00, 2.08)
	3 d	No treatment	vs.	Plasma 1 min/3 mm	0.016	No treatment > plasma 1 min/3 mm	3.49 (2.30, 4.34)
		No treatment	vs.	Plasma 1 min/10 mm	0.017	No treatment > plasma 1 min/10 mm	3.34 (1.99, 4.34)
		No treatment	vs.	Plasma 3 min/3 mm	0.012	No treatment > plasma 3 min/3 mm	4.25 (3.60, 4.70)
		No treatment	vs.	Plasma 3 min/10 mm	0.016	No treatment > plasma 3 min/10 mm	3.93 (2.39, 4.34)
		No treatment	vs.	Plasma 5 min/3 mm	0.012	No treatment > plasma 5 min/3 mm	4.25 (3.60, 4.70)
		No treatment	vs.	Plasma 5 min/10 mm	0.012	No treatment > plasma 5 min/10 mm	4.25 (3.60, 4.70)
		Chlorhexidine	vs.	Plasma 1 min/3 mm	0.238	Chlorhexidine&plasma 1 min/3 mm n.s.	1.08 (-1.32, 2.64)
		Chlorhexidine	vs.	Plasma 1 min/10 mm	0.340	Chlorhexidine&plasma 1 min/10 mm n.s.	0.65 (-1.61, 2.64)
		Chlorhexidine	vs.	Plasma 3 min/3 mm	0.051	Chlorhexidine & plasma 3 min/3 mm n.s.	2.11 (0.00, 2.64)
		Chlorhexidine	vs.	Plasma 3 min/10 mm	0.174	Chlorhexidine & plasma 3 min/10 mm n.s.	1.30 (0.00, 2.64)
		Chlorhexidine	vs.	Plasma 5 min/3 mm	0.051	Chlorhexidine & plasma 5 min/3 mm n.s.	2.11 (0.00, 2.64)
		Chlorhexidine	vs.	Plasma 5 min/10 mm	0.051	Chlorhexidine & plasma 5 min/10 mm n.s.	2.11 (0.00, 2.64)
		Amoxicillin	vs.	Plasma 1 min/3 mm	0.016	Amoxicillin > plasma 1 min/3 mm	2.76 (1.77, 3.72)
		Amoxicillin	vs.	Plasma 1 min/10 mm	0.017	Amoxicillin > plasma 1 min/10 mm	2.62 (1.39, 3.72)
		Amoxicillin	vs.	Plasma 3 min/3 mm	0.012	Amoxicillin > plasma 3 min/3 mm	3.69 (3.00, 3.85)
		Amoxicillin	vs.	Plasma 3 min/10 mm	0.016	Amoxicillin > plasma 3 min/10 mm	3.48 (1.79, 3.72)
		Amoxicillin	vs.	Plasma 5 min/3 mm	0.012	Amoxicillin > plasma 5 min/3 mm	3.69 (3.00, 3.85)
		Amoxicillin	vs.	Plasma 5 min/10 mm	0.012	Amoxicillin > plasma 5 min/10 mm	3.69 (3.00, 3.85)
		Metronidazole	vs.	Plasma 1 min/3 mm	0.016	Metronidazole > plasma 1 min/3 mm	2.58 (1.47, 3.75)
		Metronidazole	vs.	Plasma 1 min/10 mm	0.016	Metronidazole > plasma 1 min/10 mm	2.43 (1.09, 3.75)
		Metronidazole	vs.	Plasma 3 min/3 mm	0.012	Metronidazole > plasma 3 min/3 mm	3.62 (2.70, 3.78)
		Metronidazole	vs.	Plasma 3 min/10 mm	0.016	Metronidazole > plasma 3 min/10 mm	2.79 (1.71, 3.75)
		Metronidazole	vs.	Plasma 5 min/3 mm	0.012	Metronidazole > plasma 5 min/3 mm	3.62 (2.70, 3.78)
		Metronidazole	vs.	Plasma 5 min/10 mm	0.012	Metronidazole > plasma 5 min/10 mm	3.62 (2.70, 3.78)
		Amoxicillin+metronidazole	vs.	Plasma 1 min/3 mm	0.016	Amoxicillin + metronidazole > plasma 1 min	2.59 (1.39, 3.70)
		Amoxicillin+metronidazole	vs.	Plasma 1 min/10 mm	0.016	Amoxicillin + metronidazole > plasma 1 min	2.44 (1.00, 3.70)
		Amoxicillin+metronidazole	vs.	Plasma 3 min/3 mm	0.012	Amoxicillin + metronidazole > plasma 3 min	3.50 (2.70, 3.79)
		Amoxicillin+metronidazole	vs.	Plasma 3 min/10 mm	0.016	Amoxicillin + metronidazole > plasma 3 min	2.72 (1.39, 3.70)
		Amoxicillin+metronidazole	vs.	Plasma 5 min/3 mm	0.012	Amoxicillin + metronidazole > plasma 5 min	3.50 (2.70, 3.79)
		Amoxicillin+metronidazole	vs.	Plasma 5 min/10 mm	0.012	Amoxicillin + metronidazole > plasma 5 min	3.50 (2.70, 3.79)
	7 d	No treatment	vs.	Plasma 1 min/3 mm	0.012	No treatment > plasma 1 min/3 mm	4.12 (3.53, 4.89)
		No treatment	vs.	Plasma 1 min/10 mm	0.016	No treatment > plasma 1 min/10 mm	3.99 (2.62, 4.38)
		No treatment	vs.	Plasma 3 min/3 mm	0.012	No treatment > plasma 3 min/3 mm	4.12 (3.53, 4.89)
		No treatment	vs.	Plasma 3 min/10 mm	0.016	No treatment > plasma 3 min/10 mm	3.99 (2.62, 4.38)
		No treatment	vs.	Plasma 5 min/3 mm	0.014	No treatment > plasma 5 min/3 mm	4.00 (3.53, 4.38)
		No treatment	vs.	Plasma 5 min/10 mm	0.012	No treatment > plasma 5 min/10 mm	4.12 (3.53, 4.89)
		Chlorhexidine	vs.	Plasma 1 min/3 mm	0.051	Chlorhexidine & plasma 1 min/3 mm n.s.	2.05 (0.00, 2.92)
		Chlorhexidine	vs.	Plasma 1 min/10 mm	0.198	Chlorhexidine & plasma 1 min/10 mm n.s.	1.32 (0.00, 2.92)
		Chlorhexidine	vs.	Plasma 3 min/3 mm	0.051	Chlorhexidine & plasma 3 min/3 mm n.s.	2.05 (0.00, 2.92)
		Chlorhexidine	vs.	Plasma 3 min/10 mm	0.198	Chlorhexidine & plasma 3 min/10 mm n.s.	1.32 (0.00, 2.92)
		Chlorhexidine	vs.	Plasma 5 min/3 mm	0.101	Chlorhexidine & plasma 5 min/3 mm n.s.	1.39 (0.00, 2.92)
		Chlorhexidine	vs.	Plasma 5 min/10 mm	0.051	Chlorhexidine & plasma 5 min/10 mm n.s.	2.05 (0.00, 2.92)
		Amoxicillin	vs.	Plasma 1 min/3 mm	0.012	Amoxicillin > plasma 1 min/3 mm	2.80 (2.64, 3.48)
		Amoxicillin	vs.	Plasma 1 min/10 mm	0.016	Amoxicillin > plasma 1 min/10 mm	2.66 (1.32, 3.26)
		Amoxicillin	vs.	Plasma 3 min/3 mm	0.012	Amoxicillin > plasma 3 min/3 mm	2.80 (2.64, 3.48)
		Amoxicillin	vs.	Plasma 3 min/10 mm	0.016	Amoxicillin > plasma 3 min/10 mm	2.66 (1.32, 3.26)
		Amoxicillin	vs.	Plasma 5 min/3 mm	0.014	Amoxicillin > plasma 5 min/3 mm	2.68 (2.16, 3.26)
		Amoxicillin	vs.	Plasma 5 min/10 mm	0.012	Amoxicillin > plasma 5 min/10 mm	2.80 (2.64, 3.48)
		Metronidazole	vs.	Plasma 1 min/3 mm	0.025	Metronidazole > plasma 1 min/3 mm	2.84 (2.08, 3.38)
		Metronidazole	vs.	Plasma 1 min/10 mm	0.047	Metronidazole > plasma 1 min/10 mm	2.18 (0.00, 3.38)
		Metronidazole	vs.	Plasma 3 min/3 mm	0.025	Metronidazole > plasma 3 min/3 mm	2.84 (2.08, 3.38)
		Metronidazole	vs.	Plasma 3 min/10 mm	0.047	Metronidazole > plasma 3 min/10 mm	2.18 (0.00, 3.38)
		Metronidazole	vs.	Plasma 5 min/3 mm	0.036	Metronidazole > plasma 5 min/3 mm	2.82 (0.00, 3.38)
		Metronidazole	vs.	Plasma 5 min/10 mm	0.025	Metronidazole > plasma 5 min/10 mm	2.84 (2.08, 3.38)
		Amoxicillin+metronidazole	vs.	Plasma 1 min/3 mm	0.012	Amoxicillin + metronidazole > plasma 1 min/3 mm	2.81 (2.60, 3.88)
		Amoxicillin+metronidazole	vs.	Plasma 1 min/10 mm	0.016	Amoxicillin + metronidazole > plasma 1 min/10 mm	2.68 (1.30, 3.34)
		Amoxicillin+metronidazole	vs.	Plasma 3 min/3 mm	0.012	Amoxicillin + metronidazole > plasma 3 min/3 mm	2.81 (2.60, 3.88)
		Amoxicillin+metronidazole	vs.	Plasma 3 min/10 mm	0.016	Amoxicillin + metronidazole > plasma 3 min/10 mm	2.68 (1.30, 3.34)
		Amoxicillin+metronidazole	vs.	Plasma 5 min/3 mm	0.014	Amoxicillin + metronidazole > plasma 5 min/3 mm	2.70 (2.56, 3.34)
		Amoxicillin+metronidazole	vs.	Plasma 5 min/10 mm	0.012	Amoxicillin + metronidazole > plasma 5 min/10 mm	2.81 (2.60, 3.88)
*P. gingivalis*	24 h	No treatment	vs.	Plasma 1 min/3 mm	0.014	No treatment > plasma 1 min/3 mm	4.72 (3.81, 5.09)
		No treatment	vs.	Plasma 1 min/10 mm	0.016	No treatment > plasma 1 min/10 mm	4.60 (1.82, 5.09)
		No treatment	vs.	Plasma 3 min/3 mm	0.014	No treatment > plasma 3 min/3 mm	4.72 (3.52, 5.09)
		No treatment	vs.	Plasma 3 min/10 mm	0.016	No treatment > plasma 3 min/10 mm	4.60 (1.89, 5.09)
		No treatment	vs.	Plasma 5 min/3 mm	0.014	No treatment > plasma 5 min/3 mm	4.72 (3.13, 5.09)
		No treatment	vs.	Plasma 5 min/10 mm	0.012	No treatment > plasma 5 min/10 mm	4.84 (4.60, 5.13)
		Chlorhexidine	vs.	Plasma 1 min/3 mm	0.036	Chlorhexidine > plasma 1 min/3 mm	2.15 (0.00, 3.07)
		Chlorhexidine	vs.	Plasma 1 min/10 mm	0.210	Chlorhexidine & plasma 1 min/10 mm n.s.	1.79 (-0.63, 3.07)
		Chlorhexidine	vs.	Plasma 3 min/3 mm	0.036	Chlorhexidine > plasma 3 min/3 mm	2.15 (0.00, 3.07)
		Chlorhexidine	vs.	Plasma 3 min/10 mm	0.186	Chlorhexidine & plasma 3 min/10 mm n.s.	1.79 (-0.57, 3.07)
		Chlorhexidine	vs.	Plasma 5 min/3 mm	0.048	Chlorhexidine > plasma 5 min/3 mm	2.15 (0.00, 3.07)
		Chlorhexidine	vs.	Plasma 5 min/10 mm	0.025	Chlorhexidine > plasma 5 min/10 mm	2.46 (1.79, 3.07)
		Amoxicillin	vs.	Plasma 1 min/3 mm	0.014	Amoxicillin > plasma 1 min/3 mm	3.45 (2.75, 3.98)
		Amoxicillin	vs.	Plasma 1 min/10 mm	0.021	Amoxicillin > plasma 1 min/10 mm	3.38 (0.61, 3.98)
		Amoxicillin	vs.	Plasma 3 min/3 mm	0.014	Amoxicillin > plasma 3 min/3 mm	3.45 (2.46, 3.98)
		Amoxicillin	vs.	Plasma 3 min/10 mm	0.021	Amoxicillin > plasma 3 min/10 mm	3.38 (0.67, 3.98)
		Amoxicillin	vs.	Plasma 5 min/3 mm	0.014	Amoxicillin > plasma 5 min/3 mm	3.45 (2.07, 3.98)
		Amoxicillin	vs.	Plasma 5 min/10 mm	0.012	Amoxicillin > plasma 5 min/10 mm	3.66 (2.99, 4.07)
		Metronidazole	vs.	Plasma 1 min/3 mm	0.014	Metronidazole > plasma 1 min/3 mm	3.23 (2.91, 3.42)
		Metronidazole	vs.	Plasma 1 min/10 mm	0.016	Metronidazole > plasma 1 min/10 mm	3.18 (0.34, 3.42)
		Metronidazole	vs.	Plasma 3 min/3 mm	0.014	Metronidazole > plasma 3 min/3 mm	3.23 (2.62, 3.42)
		Metronidazole	vs.	Plasma 3 min/10 mm	0.016	Metronidazole > plasma 3 min/10 mm	3.18 (0.36, 3.42)
		Metronidazole	vs.	Plasma 5 min/3 mm	0.014	Metronidazole > plasma 5 min/3 mm	3.23 (2.23, 3.42)
		Metronidazole	vs.	Plasma 5 min/10 mm	0.012	Metronidazole > plasma 5 min/10 mm	3.30 (3.07, 4.23)
		Amoxicillin+metronidazole	vs.	Plasma 1 min/3 mm	0.014	Amoxicillin + metronidazole > plasma 1 min/3 mm	3.47 (3.11, 4.26)
		Amoxicillin+metronidazole	vs.	Plasma 1 min/10 mm	0.016	Amoxicillin + metronidazole > plasma 1 min/10 mm	3.28 (0.50, 4.26)
		Amoxicillin+metronidazole	vs.	Plasma 3 min/3 mm	0.014	Amoxicillin + metronidazole > plasma 3 min/3 mm	3.47 (2.82, 4.26)
		Amoxicillin+metronidazole	vs.	Plasma 3 min/10 mm	0.016	Amoxicillin + metronidazole > plasma 3 min/10 mm	3.28 (0.56, 4.26)
		Amoxicillin+metronidazole	vs.	Plasma 5 min/3 mm	0.014	Amoxicillin + metronidazole > plasma 5 min/3 mm	3.47 (2.42, 4.26)
		Amoxicillin+metronidazole	vs.	Plasma 5 min/10 mm	0.012	Amoxicillin + metronidazole > plasma 5 min/10 mm	3.67 (3.20, 4.43)
	3 d	No treatment	vs.	Plasma 1 min/3 mm	0.016	No treatment > plasma 1 min/3 mm	3.29 (2.16, 4.07)
		No treatment	vs.	Plasma 1 min/10 mm	0.017	No treatment > plasma 1 min/10 mm	2.46 (1.77, 3.93)
		No treatment	vs.	Plasma 3 min/3 mm	0.012	No treatment > plasma 3 min/3 mm	3.98 (3.78, 4.13)
		No treatment	vs.	Plasma 3 min/10 mm	0.016	No treatment > plasma 3 min/10 mm	3.29 (2.61, 4.07)
		No treatment	vs.	Plasma 5 min/3 mm	0.012	No treatment > plasma 5 min/3 mm	3.98 (3.78, 4.13)
		No treatment	vs.	Plasma 5 min/10 mm	0.012	No treatment > plasma 5 min/10 mm	3.98 (3.78, 4.13)
		Chlorhexidine	vs.	Plasma 1 min/3 mm	0.129	Chlorhexidine & plasma 1 min/3 mm n.s.	1.32 (0.00, 2.97)
		Chlorhexidine	vs.	Plasma 1 min/10 mm	0.392	Chlorhexidine & plasma 1 min/10 mm n.s.	0.68 (-1.32, 1.68)
		Chlorhexidine	vs.	Plasma 3 min/3 mm	0.025	Chlorhexidine > plasma 3 min/3 mm	2.07 (1.32, 2.97)
		Chlorhexidine	vs.	Plasma 3 min/10 mm	0.083	Chlorhexidine & plasma 3 min/10 mm n.s.	1.47 (0.00, 2.97)
		Chlorhexidine	vs.	Plasma 5 min/3 mm	0.025	Chlorhexidine > plasma 5 min/3 mm	2.07 (1.32, 2.97)
		Chlorhexidine	vs.	Plasma 5 min/10 mm	0.025	Chlorhexidine > plasma 5 min/10 mm	2.07 (1.32, 2.97)
		Amoxicillin	vs.	Plasma 1 min/3 mm	0.016	Amoxicillin > plasma 1 min/3 mm	2.78 (1.51, 3.42)
		Amoxicillin	vs.	Plasma 1 min/10 mm	0.017	Amoxicillin > plasma 1 min/10 mm	1.76 (1.07, 3.09)
		Amoxicillin	vs.	Plasma 3 min/3 mm	0.012	Amoxicillin > plasma 3 min/3 mm	3.14 (2.86, 4.02)
		Amoxicillin	vs.	Plasma 3 min/10 mm	0.016	Amoxicillin > plasma 3 min/10 mm	2.78 (1.76, 3.42)
		Amoxicillin	vs.	Plasma 5 min/3 mm	0.012	Amoxicillin > plasma 5 min/3 mm	3.14 (2.86, 4.02)
		Amoxicillin	vs.	Plasma 5 min/10 mm	0.012	Amoxicillin > plasma 5 min/10 mm	3.14 (2.86, 4.02)
		Metronidazole	vs.	Plasma 1 min/3 mm	0.016	Metronidazole > plasma 1 min/3 mm	2.48 (1.19, 3.58)
		Metronidazole	vs.	Plasma 1 min/10 mm	0.017	Metronidazole > plasma 1 min/10 mm	1.77 (0.87, 3.01)
		Metronidazole	vs.	Plasma 3 min/3 mm	0.012	Metronidazole > plasma 3 min/3 mm	3.30 (2.48, 3.85)
		Metronidazole	vs.	Plasma 3 min/10 mm	0.016	Metronidazole > plasma 3 min/10 mm	2.50 (1.69, 3.58)
		Metronidazole	vs.	Plasma 5 min/3 mm	0.012	Metronidazole > plasma 5 min/3 mm	3.30 (2.48, 3.85)
		Metronidazole	vs.	Plasma 5 min/10 mm	0.012	Metronidazole > plasma 5 min/10 mm	3.30 (2.48, 3.85)
		Amoxicillin+metronidazole	vs.	Plasma 1 min/3 mm	0.016	Amoxicillin + metronidazole > plasma 1 min	2.45 (1.28, 3.20)
		Amoxicillin+metronidazole	vs.	Plasma 1 min/10 mm	0.017	Amoxicillin + metronidazole > plasma 1 min	1.38 (0.82, 2.60)
		Amoxicillin+metronidazole	vs.	Plasma 3 min/3 mm	0.012	Amoxicillin + metronidazole > plasma 3 min	2.92 (2.60, 3.62)
		Amoxicillin+metronidazole	vs.	Plasma 3 min/10 mm	0.016	Amoxicillin + metronidazole > plasma 3 min	2.45 (1.28, 3.20)
		Amoxicillin+metronidazole	vs.	Plasma 5 min/3 mm	0.012	Amoxicillin + metronidazole > plasma 5 min	2.92 (2.60, 3.62)
		Amoxicillin+metronidazole	vs.	Plasma 5 min/10 mm	0.012	Amoxicillin + metronidazole > plasma 5 min	2.92 (2.60, 3.62)
	7 d	No treatment	vs.	Plasma 1 min/3 mm	0.012	No treatment > plasma 1 min/3 mm	3.96 (3.58, 4.28)
		No treatment	vs.	Plasma 1 min/10 mm	0.014	No treatment > plasma 1 min/10 mm	3.91 (2.96, 4.16)
		No treatment	vs.	Plasma 3 min/3 mm	0.014	No treatment > plasma 3 min/3 mm	3.91 (2.96, 4.16)
		No treatment	vs.	Plasma 3 min/10 mm	0.016	No treatment > plasma 3 min/10 mm	3.27 (2.08, 4.16)
		No treatment	vs.	Plasma 5 min/3 mm	0.014	No treatment > plasma 5 min/3 mm	3.91 (2.96, 4.16)
		No treatment	vs.	Plasma 5 min/10 mm	0.012	No treatment > plasma 5 min/10 mm	3.96 (3.58, 4.28)
		Chlorhexidine	vs.	Plasma 1 min/3 mm	0.051	Chlorhexidine & plasma 1 min/3 mm n.s.	2.38 (0.00, 2.87)
		Chlorhexidine	vs.	Plasma 1 min/10 mm	0.088	Chlorhexidine & plasma 1 min/10 mm n.s.	2.15 (0.00, 2.87)
		Chlorhexidine	vs.	Plasma 3 min/3 mm	0.088	Chlorhexidine & plasma 3 min/3 mm n.s.	2.15 (0.00, 2.87)
		Chlorhexidine	vs.	Plasma 3 min/10 mm	0.185	Chlorhexidine & plasma 3 min/10 mm n.s.	1.26 (-1.32, 2.87)
		Chlorhexidine	vs.	Plasma 5 min/3 mm	0.088	Chlorhexidine & plasma 5 min/3 mm n.s.	2.15 (0.00, 2.87)
		Chlorhexidine	vs.	Plasma 5 min/10 mm	0.051	Chlorhexidine & plasma 5 min/10 mm n.s.	2.38 (0.00, 2.87)
		Amoxicillin	vs.	Plasma 1 min/3 mm	0.012	Amoxicillin > plasma 1 min/3 mm	2.93 (2.30, 4.02)
		Amoxicillin	vs.	Plasma 1 min/10 mm	0.014	Amoxicillin > plasma 1 min/10 mm	2.78 (2.30, 3.89)
		Amoxicillin	vs.	Plasma 3 min/3 mm	0.014	Amoxicillin > plasma 3 min/3 mm	2.78 (2.30, 3.89)
		Amoxicillin	vs.	Plasma 3 min/10 mm	0.016	Amoxicillin > plasma 3 min/10 mm	2.53 (0.98, 3.89)
		Amoxicillin	vs.	Plasma 5 min/3 mm	0.014	Amoxicillin > plasma 5 min/3 mm	2.78 (2.30, 3.89)
		Amoxicillin	vs.	Plasma 5 min/10 mm	0.012	Amoxicillin > plasma 5 min/10 mm	2.93 (2.30, 4.02)
		Metronidazole	vs.	Plasma 1 min/3 mm	0.012	Metronidazole > plasma 1 min/3 mm	3.03 (2.78, 3.81)
		Metronidazole	vs.	Plasma 1 min/10 mm	0.014	Metronidazole > plasma 1 min/10 mm	2.88 (2.48, 3.66)
		Metronidazole	vs.	Plasma 3 min/3 mm	0.014	Metronidazole > plasma 3 min/3 mm	2.88 (2.48, 3.66)
		Metronidazole	vs.	Plasma 3 min/10 mm	0.016	Metronidazole > plasma 3 min/10 mm	2.63 (1.46, 3.66)
		Metronidazole	vs.	Plasma 5 min/3 mm	0.014	Metronidazole > plasma 5 min/3 mm	2.88 (2.48, 3.66)
		Metronidazole	vs.	Plasma 5 min/10 mm	0.012	Metronidazole > plasma 5 min/10 mm	3.03 (2.78, 3.81)
		Amoxicillin+metronidazole	vs.	Plasma 1 min/3 mm	0.012	Amoxicillin + metronidazole > plasma 1 min/3 mm	3.02 (2.30, 3.83)
		Amoxicillin+metronidazole	vs.	Plasma 1 min/10 mm	0.014	Amoxicillin + metronidazole > plasma 1 min/10 mm	2.98 (2.30, 3.76)
		Amoxicillin+metronidazole	vs.	Plasma 3 min/3 mm	0.014	Amoxicillin + metronidazole > plasma 3 min/3 mm	2.98 (2.30, 3.76)
		Amoxicillin+metronidazole	vs.	Plasma 3 min/10 mm	0.016	Amoxicillin + metronidazole > plasma 3 min/10 mm	2.30 (0.98, 3.76)
		Amoxicillin+metronidazole	vs.	Plasma 5 min/3 mm	0.014	Amoxicillin + metronidazole > plasma 5 min/3 mm	2.98 (2.30, 3.76)
		Amoxicillin+metronidazole	vs.	Plasma 5 min/10 mm	0.012	Amoxicillin + metronidazole > plasma 5 min/10 mm	3.02 (2.30, 3.83)
*S. oralis*	24 h	No treatment	vs.	Plasma 1 min/3 mm	0.012	No treatment > plasma 1 min/3 mm	4.63 (4.46, 4.94)
		No treatment	vs.	Plasma 1 min/10 mm	0.016	No treatment > plasma 1 min/10 mm	4.60 (1.85, 4.81)
		No treatment	vs.	Plasma 3 min/3 mm	0.012	No treatment > plasma 3 min/3 mm	4.63 (4.46, 4.94)
		No treatment	vs.	Plasma 3 min/10 mm	0.016	No treatment > plasma 3 min/10 mm	4.60 (2.20, 4.81)
		No treatment	vs.	Plasma 5 min/3 mm	0.014	No treatment > plasma 5 min/3 mm	4.60 (2.74, 4.81)
		No treatment	vs.	Plasma 5 min/10 mm	0.014	No treatment > plasma 5 min/10 mm	4.60 (3.33, 4.81)
		Chlorhexidine	vs.	Plasma 1 min/3 mm	0.102	Chlorhexidine & plasma 1 min/3 mm n.s.	0.66 (0.00, 3.42)
		Chlorhexidine	vs.	Plasma 1 min/10 mm	0.545	Chlorhexidine & plasma 1 min/10 mm n.s.	0.00 (-1.63, 3.42)
		Chlorhexidine	vs.	Plasma 3 min/3 mm	0.102	Chlorhexidine & plasma 3 min/3 mm n.s.	0.66 (0.00, 3.42)
		Chlorhexidine	vs.	Plasma 3 min/10 mm	0.545	Chlorhexidine & plasma 3 min/10 mm n.s.	0.00 (-1.28, 3.42)
		Chlorhexidine	vs.	Plasma 5 min/3 mm	0.277	Chlorhexidine & plasma 5 min/3 mm n.s.	0.00 (0.00, 3.42)
		Chlorhexidine	vs.	Plasma 5 min/10 mm	0.277	Chlorhexidine & plasma 5 min/10 mm n.s.	0.00 (0.00, 3.42)
		Amoxicillin	vs.	Plasma 1 min/3 mm	0.012	Amoxicillin > plasma 1 min/3 mm	3.83 (2.62, 4.35)
		Amoxicillin	vs.	Plasma 1 min/10 mm	0.021	Amoxicillin > plasma 1 min/10 mm	3.01 (1.15, 4.21)
		Amoxicillin	vs.	Plasma 3 min/3 mm	0.012	Amoxicillin > plasma 3 min/3 mm	3.83 (2.62, 4.35)
		Amoxicillin	vs.	Plasma 3 min/10 mm	0.016	Amoxicillin > plasma 3 min/10 mm	3.01 (1.00, 4.21)
		Amoxicillin	vs.	Plasma 5 min/3 mm	0.014	Amoxicillin > plasma 5 min/3 mm	3.55 (2.14, 4.21)
		Amoxicillin	vs.	Plasma 5 min/10 mm	0.014	Amoxicillin > plasma 5 min/10 mm	3.55 (2.62, 4.21)
		Metronidazole	vs.	Plasma 1 min/3 mm	0.012	Metronidazole > plasma 1 min/3 mm	3.24 (3.05, 4.26)
		Metronidazole	vs.	Plasma 1 min/10 mm	0.016	Metronidazole > plasma 1 min/10 mm	3.18 (0.45, 3.41)
		Metronidazole	vs.	Plasma 3 min/3 mm	0.012	Metronidazole > plasma 3 min/3 mm	3.24 (3.05, 4.26)
		Metronidazole	vs.	Plasma 3 min/10 mm	0.016	Metronidazole > plasma 3 min/10 mm	3.18 (0.81, 3.41)
		Metronidazole	vs.	Plasma 5 min/3 mm	0.014	Metronidazole > plasma 5 min/3 mm	3.22 (2.05, 3.41)
		Metronidazole	vs.	Plasma 5 min/10 mm	0.014	Metronidazole > plasma 5 min/10 mm	3.22 (2.65, 3.41)
		Amoxicillin+metronidazole	vs.	Plasma 1 min/3 mm	0.012	Amoxicillin + metronidazole > plasma 1 min/3 mm	3.83 (3.20, 4.39)
		Amoxicillin+metronidazole	vs.	Plasma 1 min/10 mm	0.016	Amoxicillin + metronidazole > plasma 1 min/10 mm	3.58 (0.93, 3.88)
		Amoxicillin+metronidazole	vs.	Plasma 3 min/3 mm	0.012	Amoxicillin + metronidazole > plasma 3 min/3 mm	3.83 (3.20, 4.39)
		Amoxicillin+metronidazole	vs.	Plasma 3 min/10 mm	0.016	Amoxicillin + metronidazole > plasma 3 min/10 mm	3.58 (1.24, 3.88)
		Amoxicillin+metronidazole	vs.	Plasma 5 min/3 mm	0.014	Amoxicillin + metronidazole > plasma 5 min/3 mm	3.82 (2.18, 3.88)
		Amoxicillin+metronidazole	vs.	Plasma 5 min/10 mm	0.014	Amoxicillin + metronidazole > plasma 5 min/10 mm	3.82 (2.77, 3.88)
	3 d	No treatment	vs.	Plasma 1 min/3 mm	0.012	No treatment > plasma 1 min/3 mm	3.95 (3.79, 4.48)
		No treatment	vs.	Plasma 1 min/10 mm	0.017	No treatment > plasma 1 min/10 mm	2.61 (1.71, 3.96)
		No treatment	vs.	Plasma 3 min/3 mm	0.012	No treatment > plasma 3 min/3 mm	3.95 (3.79, 4.48)
		No treatment	vs.	Plasma 3 min/10 mm	0.016	No treatment > plasma 3 min/10 mm	3.48 (2.10, 4.01)
		No treatment	vs.	Plasma 5 min/3 mm	0.012	No treatment > plasma 5 min/3 mm	3.95 (3.79, 4.48)
		No treatment	vs.	Plasma 5 min/10 mm	0.012	No treatment > plasma 5 min/10 mm	3.95 (3.79, 4.48)
		Chlorhexidine	vs.	Plasma 1 min/3 mm	0.025	Chlorhexidine > plasma 1 min/3 mm	2.26 (1.32, 2.99)
		Chlorhexidine	vs.	Plasma 1 min/10 mm	0.315	Chlorhexidine & plasma 1 min/10 mm n.s.	1.16 (-0.98, 2.90)
		Chlorhexidine	vs.	Plasma 3 min/3 mm	0.025	Chlorhexidine > plasma 3 min/3 mm	2.26 (1.32, 2.99)
		Chlorhexidine	vs.	Plasma 3 min/10 mm	0.129	Chlorhexidine&plasma 3 min/10 mm n.s.	1.46 (0.00, 2.99)
		Chlorhexidine	vs.	Plasma 5 min/3 mm	0.025	Chlorhexidine > plasma 5 min/3 mm	2.26 (1.32, 2.99)
		Chlorhexidine	vs.	Plasma 5 min/10 mm	0.025	Chlorhexidine > plasma 5 min/10 mm	2.26 (1.32, 2.99)
		Amoxicillin	vs.	Plasma 1 min/3 mm	0.012	Amoxicillin > plasma 1 min/3 mm	3.42 (3.10, 3.92)
		Amoxicillin	vs.	Plasma 1 min/10 mm	0.017	Amoxicillin > plasma 1 min/10 mm	2.03 (1.27, 3.42)
		Amoxicillin	vs.	Plasma 3 min/3 mm	0.012	Amoxicillin > plasma 3 min/3 mm	3.42 (3.10, 3.92)
		Amoxicillin	vs.	Plasma 3 min/10 mm	0.016	Amoxicillin > plasma 3 min/10 mm	2.85 (1.66, 3.57)
		Amoxicillin	vs.	Plasma 5 min/3 mm	0.012	Amoxicillin > plasma 5 min/3 mm	3.42 (3.10, 3.92)
		Amoxicillin	vs.	Plasma 5 min/10 mm	0.012	Amoxicillin > plasma 5 min/10 mm	3.42 (3.10, 3.92)
		Metronidazole	vs.	Plasma 1 min/3 mm	0.012	Metronidazole > plasma 1 min/3 mm	3.36 (2.48, 3.66)
		Metronidazole	vs.	Plasma 1 min/10 mm	0.017	Metronidazole > plasma 1 min/10 mm	1.95 (1.04, 3.37)
		Metronidazole	vs.	Plasma 3 min/3 mm	0.012	Metronidazole > plasma 3 min/3 mm	3.36 (2.48, 3.66)
		Metronidazole	vs.	Plasma 3 min/10 mm	0.016	Metronidazole > plasma 3 min/10 mm	2.41 (1.43, 3.64)
		Metronidazole	vs.	Plasma 5 min/3 mm	0.012	Metronidazole > plasma 5 min/3 mm	3.36 (2.48, 3.66)
		Metronidazole	vs.	Plasma 5 min/10 mm	0.012	Metronidazole > plasma 5 min/10 mm	3.36 (2.48, 3.66)
		Amoxicillin+metronidazole	vs.	Plasma 1 min/3 mm	0.012	Amoxicillin + metronidazole > plasma 1 min	3.31 (2.60, 4.34)
		Amoxicillin+metronidazole	vs.	Plasma 1 min/10 mm	0.017	Amoxicillin + metronidazole > plasma 1 min	2.02 (0.99, 3.49)
		Amoxicillin+metronidazole	vs.	Plasma 3 min/3 mm	0.012	Amoxicillin + metronidazole > Plasma 3 min	3.31 (2.60, 4.34)
		Amoxicillin+metronidazole	vs.	Plasma 3 min/10 mm	0.016	Amoxicillin + metronidazole > plasma 3 min	2.60 (1.28, 3.62)
		Amoxicillin+metronidazole	vs.	Plasma 5 min/3 mm	0.012	Amoxicillin + metronidazole > plasma 5 min	3.31 (2.60, 4.34)
		Amoxicillin+metronidazole	vs.	Plasma 5 min/10 mm	0.012	Amoxicillin + metronidazole > plasma 5 min	3.31 (2.60, 4.34)
	7 d	No treatment	vs.	Plasma 1 min/3 mm	0.012	No treatment > plasma 1 min/3 mm	4.23 (3.32, 5.31)
		No treatment	vs.	Plasma 1 min/10 mm	0.014	No treatment > plasma 1 min/10 mm	3.95 (2.56, 4.67)
		No treatment	vs.	Plasma 3 min/3 mm	0.012	No treatment > plasma 3 min/3 mm	4.23 (3.32, 5.31)
		No treatment	vs.	Plasma 3 min/10 mm	0.014	No treatment > plasma 3 min/10 mm	3.95 (3.32, 4.67)
		No treatment	vs.	Plasma 5 min/3 mm	0.012	No treatment > plasma 5 min/3 mm	4.23 (3.32, 5.31)
		No treatment	vs.	Plasma 5 min/10 mm	0.012	No treatment > plasma 5 min/10 mm	4.23 (3.32, 5.31)
		Chlorhexidine	vs.	Plasma 1 min/3 mm	0.203	Chlorhexidine & plasma 1 min/3 mm n.s.	0.00 (0.00, 3.31)
		Chlorhexidine	vs.	Plasma 1 min/10 mm	0.477	Chlorhexidine & plasma 1 min/10 mm n.s.	0.00 (0.00, 3.31)
		Chlorhexidine	vs.	Plasma 3 min/3 mm	0.203	Chlorhexidine & plasma 3 min/3 mm n.s.	0.00 (0.00, 3.31)
		Chlorhexidine	vs.	Plasma 3 min/10 mm	0.477	Chlorhexidine & plasma 3 min/10 mm n.s.	0.00 (0.00, 3.31)
		Chlorhexidine	vs.	Plasma 5 min/3 mm	0.203	Chlorhexidine & plasma 5 min/3 mm n.s.	0.00 (0.00, 3.31)
		Chlorhexidine	vs.	Plasma 5 min/10 mm	0.203	Chlorhexidine & plasma 5 min/10 mm n.s.	0.00 (0.00, 3.31)
		Amoxicillin	vs.	Plasma 1 min/3 mm	0.025	Amoxicillin > plasma 1 min/3 mm	2.31 (1.91, 3.78)
		Amoxicillin	vs.	Plasma 1 min/10 mm	0.087	Amoxicillin & plasma 1 min/10 mm n.s.	2.15 (0.00, 3.78)
		Amoxicillin	vs.	Plasma 3 min/3 mm	0.025	Amoxicillin > plasma 3 min/3 mm	2.31 (1.91, 3.78)
		Amoxicillin	vs.	Plasma 3 min/10 mm	0.036	Amoxicillin > plasma 3 min/10 mm	2.15 (0.00, 3.78)
		Amoxicillin	vs.	Plasma 5 min/3 mm	0.025	Amoxicillin > plasma 5 min/3 mm	2.31 (1.91, 3.78)
		Amoxicillin	vs.	Plasma 5 min/10 mm	0.025	Amoxicillin > plasma 5 min/10 mm	2.31 (1.91, 3.78)
		Metronidazole	vs.	Plasma 1 min/3 mm	0.051	Metronidazole & plasma 1 min/3 mm n.s.	3.25 (0.00, 3.37)
		Metronidazole	vs.	Plasma 1 min/10 mm	0.088	Metronidazole & plasma 1 min/10 mm n.s.	3.16 (0.00, 3.37)
		Metronidazole	vs.	Plasma 3 min/3 mm	0.051	Metronidazole & plasma 3 min/3 mm n.s.	3.25 (0.00, 3.37)
		Metronidazole	vs.	Plasma 3 min/10 mm	0.088	Metronidazole & plasma 3 min/10 mm n.s.	3.16 (0.00, 3.37)
		Metronidazole	vs.	Plasma 5 min/3 mm	0.051	Metronidazole & plasma 5 min/3 mm n.s.	3.25 (0.00, 3.37)
		Metronidazole	vs.	Plasma 5 min/10 mm	0.051	Metronidazole & plasma 5 min/10 mm n.s.	3.25 (0.00, 3.37)
		Amoxicillin+metronidazole	vs.	Plasma 1 min/3 mm	0.025	Amoxicillin + metronidazole > plasma 1 min/3 mm	2.64 (2.58, 3.51)
		Amoxicillin+metronidazole	vs.	Plasma 1 min/10 mm	0.086	Amoxicillin + metronidazole&plasma 1 min/10 mm n.s.	2.58 (0.00, 3.51)
		Amoxicillin+metronidazole	vs.	Plasma 3 min/3 mm	0.025	Amoxicillin + metronidazole > plasma 3 min/3 mm	2.64 (2.58, 3.51)
		Amoxicillin+metronidazole	vs.	Plasma 3 min/10 mm	0.035	Amoxicillin + metronidazole > plasma 3 min/10 mm	2.58 (0.00, 3.51)
		Amoxicillin+metronidazole	vs.	Plasma 5 min/3 mm	0.025	Amoxicillin + metronidazole > plasma 5 min/3 mm	2.64 (2.58, 3.51)
		Amoxicillin+metronidazole	vs.	Plasma 5 min/10 mm	0.025	Amoxicillin + metronidazole > plasma 5 min/10 mm	2.64 (2.58, 3.51)
*V. dispar*	24 h	No treatment	vs.	Plasma 1 min/3 mm	0.012	No treatment > plasma 1 min/3 mm	4.58 (4.00, 5.00)
		No treatment	vs.	Plasma 1 min/10 mm	0.016	No treatment > plasma 1 min/10 mm	4.32 (2.64, 4.85)
		No treatment	vs.	Plasma 3 min/3 mm	0.012	No treatment > plasma 3 min/3 mm	4.58 (4.00, 5.00)
		No treatment	vs.	Plasma 3 min/10 mm	0.016	No treatment > plasma 3 min/10 mm	4.32 (2.35, 4.85)
		No treatment	vs.	Plasma 5 min/3 mm	0.014	No treatment > plasma 5 min/3 mm	4.56 (3.68, 4.85)
		No treatment	vs.	Plasma 5 min/10 mm	0.012	No treatment > plasma 5 min/10 mm	4.58 (4.00, 5.00)
		Chlorhexidine	vs.	Plasma 1 min/3 mm	0.012	Chlorhexidine > plasma 1 min/3 mm	2.61 (2.21, 2.86)
		Chlorhexidine	vs.	Plasma 1 min/10 mm	0.018	Chlorhexidine > plasma 1 min/10 mm	2.26 (0.65, 2.68)
		Chlorhexidine	vs.	Plasma 3 min/3 mm	0.012	Chlorhexidine > plasma 3 min/3 mm	2.61 (2.21, 2.86)
		Chlorhexidine	vs.	Plasma 3 min/10 mm	0.018	Chlorhexidine > plasma 3 min/10 mm	2.26 (0.48, 2.68)
		Chlorhexidine	vs.	Plasma 5 min/3 mm	0.014	Chlorhexidine > plasma 5 min/3 mm	2.53 (1.54, 2.68)
		Chlorhexidine	vs.	Plasma 5 min/10 mm	0.012	Chlorhexidine > plasma 5 min/10 mm	2.61 (2.21, 2.86)
		Amoxicillin	vs.	Plasma 1 min/3 mm	0.012	Amoxicillin > plasma 1 min/3 mm	2.53 (2.08, 4.56)
		Amoxicillin	vs.	Plasma 1 min/10 mm	0.021	Amoxicillin > plasma 1 min/10 mm	2.38 (0.76, 3.75)
		Amoxicillin	vs.	Plasma 3 min/3 mm	0.012	Amoxicillin > plasma 3 min/3 mm	2.53 (2.08, 4.56)
		Amoxicillin	vs.	Plasma 3 min/10 mm	0.021	Amoxicillin > plasma 3 min/10 mm	2.33 (0.30, 3.75)
		Amoxicillin	vs.	Plasma 5 min/3 mm	0.014	Amoxicillin > plasma 5 min/3 mm	2.42 (2.08, 3.75)
		Amoxicillin	vs.	Plasma 5 min/10 mm	0.012	Amoxicillin > plasma 5 min/10 mm	2.53 (2.08, 4.56)
		Metronidazole	vs.	Plasma 1 min/3 mm	0.012	Metronidazole > plasma 1 min/3 mm	2.60 (2.30, 4.82)
		Metronidazole	vs.	Plasma 1 min/10 mm	0.016	Metronidazole > plasma 1 min/10 mm	2.48 (0.98, 3.72)
		Metronidazole	vs.	Plasma 3 min/3 mm	0.012	Metronidazole > plasma 3 min/3 mm	2.60 (2.30, 4.82)
		Metronidazole	vs.	Plasma 3 min/10 mm	0.016	Metronidazole > plasma 3 min/10 mm	2.48 (0.44, 3.72)
		Metronidazole	vs.	Plasma 5 min/3 mm	0.014	Metronidazole > plasma 5 min/3 mm	2.51 (2.30, 3.72)
		Metronidazole	vs.	Plasma 5 min/10 mm	0.012	Metronidazole > plasma 5 min/10 mm	2.60 (2.30, 4.82)
		Amoxicillin+metronidazole	vs.	Plasma 1 min/3 mm	0.012	Amoxicillin + metronidazole > plasma 1 min/3 mm	2.58 (1.79, 4.06)
		Amoxicillin+metronidazole	vs.	Plasma 1 min/10 mm	0.021	Amoxicillin + metronidazole > plasma 1 min/10 mm	2.38 (0.46, 3.93)
		Amoxicillin+metronidazole	vs.	Plasma 3 min/3 mm	0.012	Amoxicillin + metronidazole > plasma 3 min/3 mm	2.58 (1.79, 4.06)
		Amoxicillin+metronidazole	vs.	Plasma 3 min/10 mm	0.028	Amoxicillin + metronidazole > plasma 3 min/10 mm	2.26 (0.30, 3.93)
		Amoxicillin+metronidazole	vs.	Plasma 5 min/3 mm	0.014	Amoxicillin + metronidazole > plasma 5 min/3 mm	2.51 (1.79, 3.93)
		Amoxicillin+metronidazole	vs.	Plasma 5 min/10 mm	0.012	Amoxicillin + metronidazole > plasma 5 min/10 mm	2.58 (1.79, 4.06)
	3 d	No treatment	vs.	Plasma 1 min/3 mm	0.012	No treatment > plasma 1 min/3 mm	3.92 (3.15, 4.41)
		No treatment	vs.	Plasma 1 min/10 mm	0.017	No treatment > plasma 1 min/10 mm	2.97 (2.01, 4.02)
		No treatment	vs.	Plasma 3 min/3 mm	0.012	No treatment > plasma 3 min/3 mm	3.92 (3.15, 4.41)
		No treatment	vs.	Plasma 3 min/10 mm	0.016	No treatment > plasma 3 min/10 mm	3.79 (2.59, 4.02)
		No treatment	vs.	Plasma 5 min/3 mm	0.012	No treatment > plasma 5 min/3 mm	3.92 (3.15, 4.41)
		No treatment	vs.	Plasma 5 min/10 mm	0.012	No treatment > plasma 5 min/10 mm	3.92 (3.15, 4.41)
		Chlorhexidine	vs.	Plasma 1 min/3 mm	0.102	Chlorhexidine & plasma 1 min/3 mm n.s.	0.81 (0.00, 2.00)
		Chlorhexidine	vs.	Plasma 1 min/10 mm	0.802	Chlorhexidine & plasma 1 min/10 mm n.s.	0.00 (-1.79, 2.00)
		Chlorhexidine	vs.	Plasma 3 min/3 mm	0.102	Chlorhexidine & plasma 3 min/3 mm n.s.	0.81 (0.00, 2.00)
		Chlorhexidine	vs.	Plasma 3 min/10 mm	0.346	Chlorhexidine & plasma 3 min/10 mm n.s.	0.15 (0.00, 2.00)
		Chlorhexidine	vs.	Plasma 5 min/3 mm	0.102	Chlorhexidine & plasma 5 min/3 mm n.s.	0.81 (0.00, 2.00)
		Chlorhexidine	vs.	Plasma 5 min/10 mm	0.102	Chlorhexidine & plasma 5 min/10 mm n.s.	0.81 (0.00, 2.00)
		Amoxicillin	vs.	Plasma 1 min/3 mm	0.012	Amoxicillin > plasma 1 min/3 mm	2.84 (2.15, 3.42)
		Amoxicillin	vs.	Plasma 1 min/10 mm	0.017	Amoxicillin > plasma 1 min/10 mm	1.98 (0.54, 3.34)
		Amoxicillin	vs.	Plasma 3 min/3 mm	0.012	Amoxicillin > plasma 3 min/3 mm	2.84 (2.15, 3.42)
		Amoxicillin	vs.	Plasma 3 min/10 mm	0.016	Amoxicillin > plasma 3 min/10 mm	2.26 (1.28, 3.34)
		Amoxicillin	vs.	Plasma 5 min/3 mm	0.012	Amoxicillin > plasma 5 min/3 mm	2.84 (2.15, 3.42)
		Amoxicillin	vs.	Plasma 5 min/10 mm	0.012	Amoxicillin > plasma 5 min/10 mm	2.84 (2.15, 3.42)
		Metronidazole	vs.	Plasma 1 min/3 mm	0.012	Metronidazole > plasma 1 min/3 mm	2.57 (1.61, 2.89)
		Metronidazole	vs.	Plasma 1 min/10 mm	0.034	Metronidazole > plasma 1 min/10 mm	1.45 (0.22, 2.78)
		Metronidazole	vs.	Plasma 3 min/3 mm	0.012	Metronidazole > plasma 3 min/3 mm	2.57 (1.61, 2.89)
		Metronidazole	vs.	Plasma 3 min/10 mm	0.016	Metronidazole > plasma 3 min/10 mm	2.00 (1.13, 2.78)
		Metronidazole	vs.	Plasma 5 min/3 mm	0.012	Metronidazole > plasma 5 min/3 mm	2.57 (1.61, 2.89)
		Metronidazole	vs.	Plasma 5 min/10 mm	0.012	Metronidazole > plasma 5 min/10 mm	2.57 (1.61, 2.89)
		Amoxicillin+metronidazole	vs.	Plasma 1 min/3 mm	0.012	Amoxicillin + metronidazole > plasma 1 min	2.90 (2.38, 3.85)
		Amoxicillin+metronidazole	vs.	Plasma 1 min/10 mm	0.016	Amoxicillin + metronidazole > plasma 1 min	2.31 (0.77, 3.00)
		Amoxicillin+metronidazole	vs.	Plasma 3 min/3 mm	0.012	Amoxicillin + metronidazole > plasma 3 min	2.90 (2.38, 3.85)
		Amoxicillin+metronidazole	vs.	Plasma 3 min/10 mm	0.015	Amoxicillin + metronidazole > plasma 3 min	2.51 (1.58, 3.00)
		Amoxicillin+metronidazole	vs.	Plasma 5 min/3 mm	0.012	Amoxicillin + metronidazole > plasma 5 min	2.90 (2.38, 3.85)
		Amoxicillin+metronidazole	vs.	Plasma 5 min/10 mm	0.012	Amoxicillin + metronidazole > plasma 5 min	2.90 (2.38, 3.85)
	7 d	No treatment	vs.	Plasma 1 min/3 mm	0.012	No treatment > plasma 1 min/3 mm	3.89 (2.90, 4.81)
		No treatment	vs.	Plasma 1 min/10 mm	0.012	No treatment > plasma 1 min/10 mm	3.89 (2.90, 4.81)
		No treatment	vs.	Plasma 3 min/3 mm	0.012	No treatment > plasma 3 min/3 mm	3.89 (2.90, 4.81)
		No treatment	vs.	Plasma 3 min/10 mm	0.012	No treatment > plasma 3 min/10 mm	3.89 (2.90, 4.81)
		No treatment	vs.	Plasma 5 min/3 mm	0.012	No treatment > plasma 5 min/3 mm	3.89 (2.90, 4.81)
		No treatment	vs.	Plasma 5 min/10 mm	0.012	No treatment > plasma 5 min/10 mm	3.89 (2.90, 4.81)
		Chlorhexidine	vs.	Plasma 1 min/3 mm	0.102	Chlorhexidine & plasma 1 min/3 mm n.s.	0.81 (0.00, 2.08)
		Chlorhexidine	vs.	Plasma 1 min/10 mm	0.102	Chlorhexidine & plasma 1 min/10 mm n.s.	0.81 (0.00, 2.08)
		Chlorhexidine	vs.	Plasma 3 min/3 mm	0.102	Chlorhexidine & plasma 3 min/3 mm n.s.	0.81 (0.00, 2.08)
		Chlorhexidine	vs.	Plasma 3 min/10 mm	0.102	Chlorhexidine & plasma 3 min/10 mm n.s.	0.81 (0.00, 2.08)
		Chlorhexidine	vs.	Plasma 5 min/3 mm	0.102	Chlorhexidine & plasma 5 min/3 mm n.s.	0.81 (0.00, 2.08)
		Chlorhexidine	vs.	Plasma 5 min/10 mm	0.102	Chlorhexidine & plasma 5 min/10 mm n.s.	0.81 (0.00, 2.08)
		Amoxicillin	vs.	Plasma 1 min/3 mm	0.025	Amoxicillin > plasma 1 min/3 mm	2.54 (1.32, 3.01)
		Amoxicillin	vs.	Plasma 1 min/10 mm	0.025	Amoxicillin > plasma 1 min/10 mm	2.54 (1.32, 3.01)
		Amoxicillin	vs.	Plasma 3 min/3 mm	0.025	Amoxicillin > plasma 3 min/3 mm	2.54 (1.32, 3.01)
		Amoxicillin	vs.	Plasma 3 min/10 mm	0.025	Amoxicillin > plasma 3 min/10 mm	2.54 (1.32, 3.01)
		Amoxicillin	vs.	Plasma 5 min/3 mm	0.025	Amoxicillin > plasma 5 min/3 mm	2.54 (1.32, 3.01)
		Amoxicillin	vs.	Plasma 5 min/10 mm	0.025	Amoxicillin > plasma 5 min/10 mm	2.54 (1.32, 3.01)
		Metronidazole	vs.	Plasma 1 min/3 mm	0.025	Metronidazole > plasma 1 min/3 mm	2.23 (1.91, 2.60)
		Metronidazole	vs.	Plasma 1 min/10 mm	0.025	Metronidazole > plasma 1 min/10 mm	2.23 (1.91, 2.60)
		Metronidazole	vs.	Plasma 3 min/3 mm	0.025	Metronidazole > plasma 3 min/3 mm	2.23 (1.91, 2.60)
		Metronidazole	vs.	Plasma 3 min/10 mm	0.025	Metronidazole > plasma 3 min/10 mm	2.23 (1.91, 2.60)
		Metronidazole	vs.	Plasma 5 min/3 mm	0.025	Metronidazole > plasma 5 min/3 mm	2.23 (1.91, 2.60)
		Metronidazole	vs.	Plasma 5 min/10 mm	0.025	Metronidazole > plasma 5 min/10 mm	2.23 (1.91, 2.60)
		Amoxicillin+metronidazole	vs.	Plasma 1 min/3 mm	0.025	Amoxicillin + metronidazole > plasma 1 min/3 mm	2.38 (1.32, 2.64)
		Amoxicillin+metronidazole	vs.	Plasma 1 min/10 mm	0.025	Amoxicillin + metronidazole > plasma 1 min/10 mm	2.38 (1.32, 2.64)
		Amoxicillin+metronidazole	vs.	Plasma 3 min/3 mm	0.025	Amoxicillin + metronidazole > plasma 3 min/3 mm	2.38 (1.32, 2.64)
		Amoxicillin+metronidazole	vs.	Plasma 3 min/10 mm	0.025	Amoxicillin + metronidazole > plasma 3 min/10 mm	2.38 (1.32, 2.64)
		Amoxicillin+metronidazole	vs.	Plasma 5 min/3 mm	0.025	Amoxicillin + metronidazole > plasma 5 min/3 mm	2.38 (1.32, 2.64)
		Amoxicillin+metronidazole	vs.	Plasma 5 min/10 mm	0.025	Amoxicillin + metronidazole > plasma 5 min/10 mm	2.38 (1.32, 2.64)

## Data Availability

The raw data used to support the findings of this study are available from the corresponding author upon request.

## Additional Points

*One-sentence summary.* In this study, we showed that a short distance and period of exposure to LTP are effective against a peri-implantitis-related biofilm formed on titanium surfaces.
